# Recent Advances in Antigen-Specific Immunotherapies for the Treatment of Multiple Sclerosis

**DOI:** 10.3390/brainsci10060333

**Published:** 2020-05-29

**Authors:** Olga Kammona, Costas Kiparissides

**Affiliations:** 1 Chemical Process and Energy Resources Institute, Centre for Research and Technology Hellas, P.O. Box 60361, 57001 Thessaloniki, Greece; kammona@cperi.certh.gr; 2 Department of Chemical Engineering, Aristotle University of Thessaloniki, 54124 Thessaloniki, Greece

**Keywords:** multiple sclerosis, autoimmune diseases, antigen-specific immunotherapies, tolerogenic vaccines, tolerance induction, central nervous system, myelin peptides, myelin basic protei, proteolipid protein, myelin oligodendrocyte glycoprotein

## Abstract

Multiple sclerosis (MS) is an autoimmune disease of the central nervous system and is considered to be the leading non-traumatic cause of neurological disability in young adults. Current treatments for MS comprise long-term immunosuppressant drugs and disease-modifying therapies (DMTs) designed to alter its progress with the enhanced risk of severe side effects. The Holy Grail for the treatment of MS is to specifically suppress the disease while at the same time allow the immune system to be functionally active against infectious diseases and malignancy. This could be achieved via the development of immunotherapies designed to specifically suppress immune responses to self-antigens (e.g., myelin antigens). The present study attempts to highlight the various antigen-specific immunotherapies developed so far for the treatment of multiple sclerosis (e.g., vaccination with myelin-derived peptides/proteins, plasmid DNA encoding myelin epitopes, tolerogenic dendritic cells pulsed with encephalitogenic epitopes of myelin proteins, attenuated autologous T cells specific for myelin antigens, T cell receptor peptides, carriers loaded/conjugated with myelin immunodominant peptides, etc.), focusing on the outcome of their recent preclinical and clinical evaluation, and to shed light on the mechanisms involved in the immunopathogenesis and treatment of multiple sclerosis.

## 1. Introduction

Multiple sclerosis (MS) is a chronic inflammatory disease of the central nervous system (CNS) caused by genetically-predisposed hosts by infectious and environmental factors which induce complex autoimmune responses in the CNS resulting in degeneration of the myelin sheath and axonal loss in the brain and spinal cord [1,2,3,4,5,6,7,8,9,10,11,12,13,14] It is the most prominent demyelinating disease leading to progressive clinical disability in MS patients [5,6,15] due to ineffective remyelination [13,15]. More than 2 million people worldwide suffer from MS and it is considered as the leading non-traumatic cause of neurological disability in young adults with a disease onset commonly around 20 and 40 years of age [4,6,15,16]. High prevalence of the disease is reported in North America and Europe [15].

MS exhibits a vastly heterogeneous clinical course [6,17] which varies from a benign disease course that doesn’t lead to serious disability, demonstrated by 10–15% of MS patients, to aggressive forms of the disease leading to severe disability and even paralysis. The increased heterogeneity of the disease severity strongly affects the design and duration of therapeutic schemes administered to MS patients [17].

**Figure 1:**
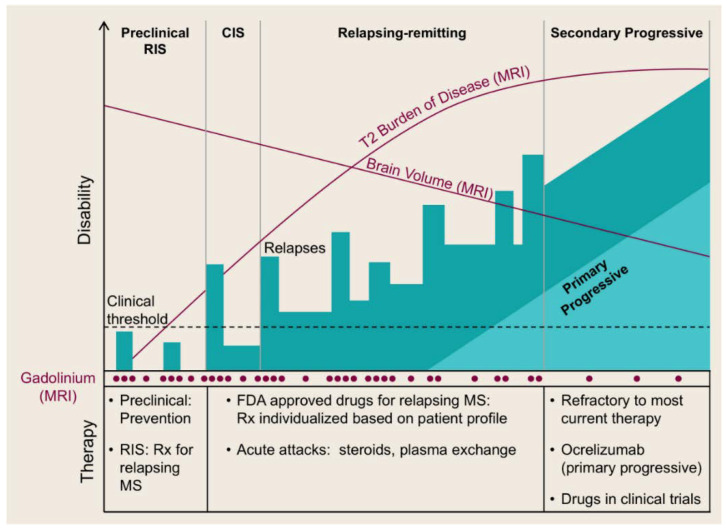
Stages of multiple sclerosis (MS). RIS: radiologically isolated syndrome; CIS: clinically isolated syndrome; FDA: U.S. food and drug administration (with the permission of [2]).

MS features the following stages: a pre-clinical stage, namely, a radiologically-isolated syndrome (RIS), which is then demonstrated as a clinically-isolated syndrome (CIS) [2,3], followed by a relapsing remitting stage (RRMS) which may later advance into secondary progressive disease (SPMS) [2,4,6,16,18]. It should be noted that a minority of MS patients (e.g., 10–15% [3,6,16]) exhibit progressive MS from the disease onset, known as primary progressive MS (PPMS) [2,4,6,18] (Figure 1). The aforementioned classification corresponds to the inflammatory image of MS which can be detected via magnetic resonance imaging (MRI) [2,16].

RRMS affects approximately 85% of MS patients [3,6,19] of whom women are twice as many as men [6]. It is characterized by periods of relapses (i.e., episodes of neurologic dysfunction, such as sensory disturbances, optic neuritis, or disturbances of motor/cerebellar function) followed by remission periods (i.e., periods of partial or full clinical recovery) [2,3,6,14,16]. Relapses coincide with CNS inflammation/demyelination visualized by MRI as lesions found mainly in the white matter [3]. In the majority of patients, RRMS advances to SPMS [16] within 10–20 years after diagnosis [3,6].

RRMS involves the movement of immune cells from the peripheral sites to the CNS (mainly in the white matter, even though extensive number of demyelinated plaques can be located in the grey matter [20]) resulting in the formation of localized inflammatory sites. Inflammatory processes in these sites induce killing of oligodendrocytes, myelin damage, and axon injury and loss, resulting in impaired neurological function [20]. On the other hand, the progressive disease implicates the generation of a pathological process within the brain [2]. Thus, the characteristic feature of SPMS is no longer the inflammatory lesions but an atrophic brain attributed to enhanced loss of axons, cortical demyelination, activation of microglia, and inefficient remyelination [2,3]. SPMS patients demonstrate progressive neurological dysfunction resulting in enhanced physical disability (e.g., inability to walk) [2,3].

PPMS is also characterized by gradual neurological decline without relapses [3,6]. In comparison with RRMS, the disease onset for PPMS is usually ten years later and it does not exhibit female predominance [6]. To date, clinical evidence shows significant differences between RRMS and progressive MS [21], reflected by the diverse response to currently existing treatments, but not between SPMS and PPMS. [18].

Currently, there is no cure for MS. Some existing treatments appear to be beneficial for patients with RRMS. However, there is still a lack of effective therapies for the progressive forms of MS [2].

The present paper aims to extensively review the different, recently developed myelin antigen-specific strategies (e.g., myelin peptide based vaccination, vaccination with plasmid DNA encoding myelin epitopes, tolerogenic dendritic cells pulsed with encephalitogenic epitopes of myelin proteins, vaccination with attenuated autologous T cells specific for myelin antigens, T cell receptor vaccination, carrier-aided administration of myelin immunodominant peptides, etc.) for the prevention/treatment of MS, especially with respect to their in vivo and clinical evaluation outcomes and the challenges they face in order to be translated to MS patients. It also seeks to unravel the mechanisms involved in the immunopathogenesis of the relapsing remitting and progressive MS, as well as the mechanisms of action of the developed tolerance-inducing vaccines.

The different antigen-specific immunotherapies are analytically presented in a comparative manner in separate tables providing detailed information about the selected myelin antigen, the vaccination strategy (e.g., prophylactic, preclinical, therapeutic), the administration route (e.g., intravenous, subcutaneous, intraperitoneal, epicutaneous, intradermal, oral, nasal, pulmonary) and the administered dose, the cell type (e.g., tolerogenic dendritic cells, T cells, hematopoietic stem cells, bone marrow cells) and the inductive agent, the carrier type (e.g., polymer particles, soluble antigen arrays, immune polyelectrolyte multilayers, inorganic particles, pMHC-NPs, mannan-conjugated myelin peptides, liposomes, exosomes, antigen-presenting yeast cells), and its characteristics (e.g., size, zeta potential, antigen loading), as well as the vaccination outcome. 

The review paper is based on a systematic search of PubMed using the following search terms: multiple sclerosis, antigen-specific immunotherapies, tolerogenic vaccines, nanocarriers, nanomedicine, DNA vaccination, cell-based vaccination, clinical trials. The search covered the time period from 1 January 2000 till today. Publications addressing pre-clinical and clinical evaluation of antigen-specific immunotherapies for multiple sclerosis were selected for inclusion.

## 2. Immunopathogenesis of MS

Successful preclinical studies and clinical trials for MS which target cells and molecules of the immune system support the idea that the latter has a dominant role in the pathogenesis of MS. These studies have proposed that cells of the adaptive immune system like B cells and various effector T cells, combined with cells of the innate immune system such as natural killer cells and microglia, uniquely contribute to the disease [2]. However, it should be mentioned that while the peripheral adaptive immune system (T lymphocytes) is the primary driver of RRMS, the innate immune system (microglia and astrocytes) together with B lymphocytes is considered to drive progressive MS [2]. The CNS of MS patients has been also found to exhibit infiltration of activated T cells, B cells, plasma cells, dendritic cells (DCs), and macrophages indicating the contribution of both cellular and humoral (i.e., antibody-mediated) immune responses as well as of various immunopathological effector mechanisms to the damage of CNS tissue [22,23]. 

It has been suggested that two independent types of inflammation, developing in parallel, can occur in multiple sclerosis patients. The first one is related with the focal invasion of T and B cells through BBB leakage, giving rise to classic active demyelinated plaques in the white matter. The second one deals with a slow accumulation of T and B lymphocytes without profound BBB damage in the perivascular Virchow Robin spaces and the meninges, where they form cellular aggregates resembling, in most severe cases, tertiary lymph follicles. The latter can be linked with the development of demyelinated lesions in the cerebral and cerebellar cortex, slow expansion of existing lesions in the white matter, and diffuse neurodegeneration in normal-appearing white and/or grey matter [18]. The presence of the lymphoid follicle-like structures (follicle-like ectopic germinal centers) in the inflamed cerebral meninges of some SPMS patients could indicate that B-cell maturation is sustained locally in the CNS and contributes to the induction of a compartmentalized humoral immune response [2,22].

The role of the various immune cells and the immunopathological effector mechanisms contributing to the development of MS are discussed below. 

**Figure 2:**
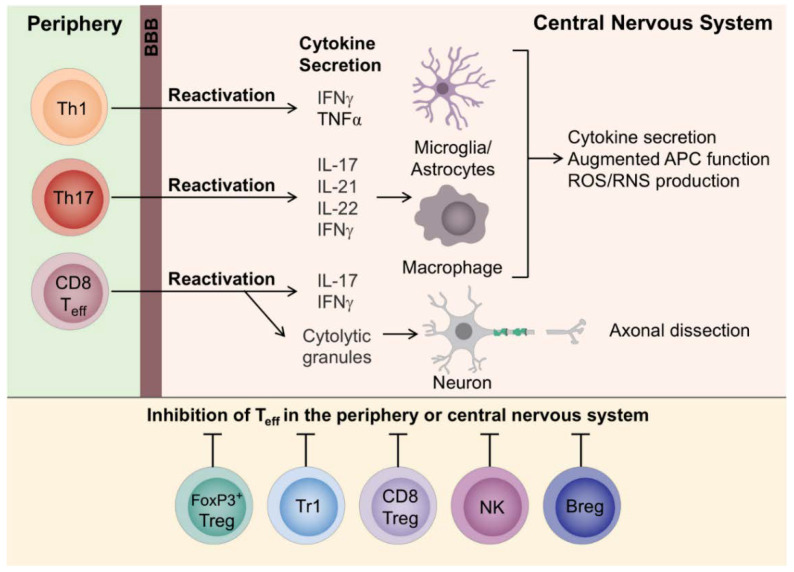
Effector T cells in multiple sclerosis (with the permission of [2]).

The ability of the human immune system to respond to an enormous number of encountered antigens comes with the risk that some T cells will be able to recognize self-antigens, such as CNS (e.g., myelin) antigens. Most autoreactive T lymphocytes are usually deleted in the thymus via a process known as negative selection (central tolerance). However, a number of these T cells escape from the thymus to peripheral sites where they are normally kept under control by mechanisms of peripheral tolerance. If these mechanisms fail, due to reduced action of regulatory T cells and/or enhanced resistance of effector T and B lymphocytes to suppression, autoreactive T cells recognizing CNS antigens are activated in the peripheral lymphoid system to become effector cells, via molecular mimicry (i.e., activation by a viral peptide having sufficient sequence similarity [24] or otherwise sharing an immunologic epitope [25] with the CNS antigen), recognition of CNS proteins released in the periphery, presentation of new autoantigens and bystander activation (i.e., T cell receptor (TCR)-independent and cytokine-dependent activation probably due to viral infection [26]). Then the activated T cells (CD8+ T cells, and CD4+ T cells differentiate to T helper 1 (Th1) and Th17 cells) together with B cells and monocytes (cells of the innate immune system) infiltrate the CNS by crossing the blood-brain barrier (BBB) leading to inflammation. There, they are reactivated via encountered resident antigen presenting cells, APCs (e.g., microglial cells) and infiltrating APCs (e.g., dendritic cells, macrophages) presenting CNS autoantigens on the major histocompatibility complex, MHC (also known as human leucocyte antigen, HLA, in humans [11]) molecules. Specifically, CD4+ T cells interact with MHC II expressing cells, like dendritic cells, macrophages and B cells, whereas CD8+ T cells directly interact with MHC I/antigen-expressing cells, like neurons and oligodendrocytes. It should be noted that MHC class II is adequately expressed only on professional APCs, while MHC class I is expressed by all cell types in the CNS inflammatory milieu. Therefore, CD4+ T cells are mainly found in perivascular cuffs, and meninges, whereas CD8+ T cells additionally infiltrate the parenchyma of the irritated lesions. Upon contact with their cognate antigen, CD4+ T cells are thought to secrete cytokines and immune mediators resulting in the attraction of resident immune cells like microglia, macrophages and astrocytes, secretion of proinflammatory cytokines, enhanced APC function, and increased production of reactive oxygen and nitrogen species (ROS/RNS). On the other hand, apart from secreting inflammatory mediators, CD8+ T cells directly attack oligodendrocytes and neurons, thus causing oligodendrocyte death (e.g., via secretion of granzymes and perforin leading to pore formation and stimulation of programmed cell death [2]) and neuronal damage (e.g., release of cytolytic granules leading to axonal dissection [2]) (Figure 2). The above result in inflammation, myelin loss, and axonal injury. This inflammatory cascade leads to the recruitment of monocytes and macrophages into the lesion resulting in the release of more CNS antigens and their presentation to potentially autoreactive T cells. It should be mentioned that epitope spreading could result in a broader autoimmune response involving additional autoantigens [1,2,3,11,27,28,29,30,31,32,33].

CD4+ T cells are considered to have a paramount role in the immunopathogenesis of MS due to the secretion of interferon gamma (IFNγ) and IL-17 [2,20,34]. However, it has been lately revealed that CD8+ T cells are also responsible for the initiation of human MS pathogenesis where, contrary to experimental autoimune encephalomyelitis (EAE), CD8+ T cells are the predominant T lymphocyte infiltrate in acute and chronic MS lesions [1,2]. Compared with CD4+ T cells, CD8+ T cells can be found more frequently in the white matter and in the cortical demyelinating lesions in the grey matter, and their density can be closely correlated with axonal damage [1,3]. Epitope spreading, assisted by cross-presentation of antigens by monocyte-derived DCs, has been found to activate myelin-specific CD8+ T cells also in an EAE model [3]. It has been suggested that CD8+ T cells remain in the CNS (e.g., brain and spinal cord) as tissue-resident cells, and upon re-encounter of their cognate antigen, focally propagate neuroinflammation [18].

Despite the fact that MS is considered a T lymphocyte-mediated disease [35], the important results of anti-CD20 therapy (e.g., rituximab, ocrelizumab) in MS indicate a significant role for B cells in its pathogenesis. B cells can have either a pro- or an anti-inflammatory role, based on their subtype and context. Their pro-inflammatory functions, comprise critical antigen presentation in the context of MHC class II molecules to Th17 and Th1 cells, secretion of pro-inflammatory cytokines (e.g., tumor necrosis factor alpha, TNF-α, interleukin-6 (IL-6) and granulocyte-macrophage colony-stimulating factor, GM-CSF) that promote CNS inflammation and propagate demyelination and neurodegeneration, and production of antibodies [36]. B lymphocytes can traffic out of the CNS to the cervical lymph nodes where they can undergo affinity maturation and then re-enter the CNS and promote further damage [3].

B cells are considered a unique population of APCs since, in contrast to other APCs which recognize various exogenous and endogenous antigens, B cells are highly selective (i.e., they specifically recognize only the antigens that are bound to their unique surface B cell receptor). Studies with the EAE model have indicated that some autoantigens, like the highly immunogenic myelin oligodendrocyte glycoprotein (MOG), require their presentation by B cells to activate CD4+ T cells. Accordingly, it can be speculated that the antigen(s) which trigger human MS are likewise B cell dependent [36]. Furthermore, active genes in B cells represent a major component of more than 200 variants known to increase the risk for developing MS. Remarkably, the gene that encodes the MHC class II DR β chain, which is known to be critical for APC function, is considered, genome-wide, the strongest MS predisposition signal. Probably, the net effect of this genetic burden is biased biology of B cells towards a pro-inflammatory phenotype, which promotes the presentation of self-antigens to effector T cells or augments the autoimmune responses through the production of cytokines and other immune mediators [36].

Regulatory T cells (CD4 FoxP3+ Tregs, CD4+ Tr1 regulatory cells, CD8 Tregs), regulatory B cells (Breg) cells and natural killer cells (NK cells) can achieve regulation of effector T cells in the peripheral lymphoid tissue or in the CNS. CD4 FoxP3+ Tregs (<4% of circulating CD4 T cells) express the transcription factor Forkhead box protein 3 (FoxP3) along with numerous inhibitory checkpoint molecules on their surface. They are activated by self-antigens and they suppress the activation of other cell types through a mechanism that requires cell contact [37]. CD4+ Tr1 regulatory cells impede cell proliferation mainly via the secretion of IL-10 [38]. Both Tregs are considered important in MS due to the exhibition of unique characteristics. Subsets of CD8+ Tregs that have been indicated to suppress immune responses and disease progression via distinct mechanisms have been identified by a unique expression of molecules like CD122, CD28, CD102 and HLA-G [2,39,40]. In addition, Th2 cells secreting cytokines like IL-4, IL-5, and IL-13, are considered to be able to downregulate the activity of pro-inflammatory cells [27]. B cells can also regulate various B and T cell mediated effector immune functions via secretion of regulatory cytokines IL-10 and IL-35, transforming growth factor beta (TGF-β), or programmed death-ligand 1 (PD-L1). Specifically, IL-10 secreting B-regs inhibit pro-inflammatory T cell responses, partly mediated via IFNγ and IL17 [2,3,36]. Finally, NK cells are known to suppress immune responses via killing activated, possibly pathogenic, CD4+ T cells. 

Immune-modulatory networks are triggered in parallel with the deleterious activity of effector T cells, in order to limit CNS inflammation and initiate tissue repair, resulting in partial remyelination. The modulation of immune activation can be associated with clinical remission. However, it should be mentioned that in the absence of treatment, suppression of autoimmunity cannot be fully achieved. Consequently, additional attacks will normally lead to the progressive form of MS [2]. The action of autoreactive T and B cells in MS could be owed to the defective function of regulatory cells. Disease-associated HLA class II variants might skew the selection in the thymus so that the regulatory T cells which are released into the peripheral sites cannot adequately suppress autoreactive effector T cells [3]. 

## 3. MS Therapies

### 3.1. Disease-Modifying Therapies

**Figure 3:**
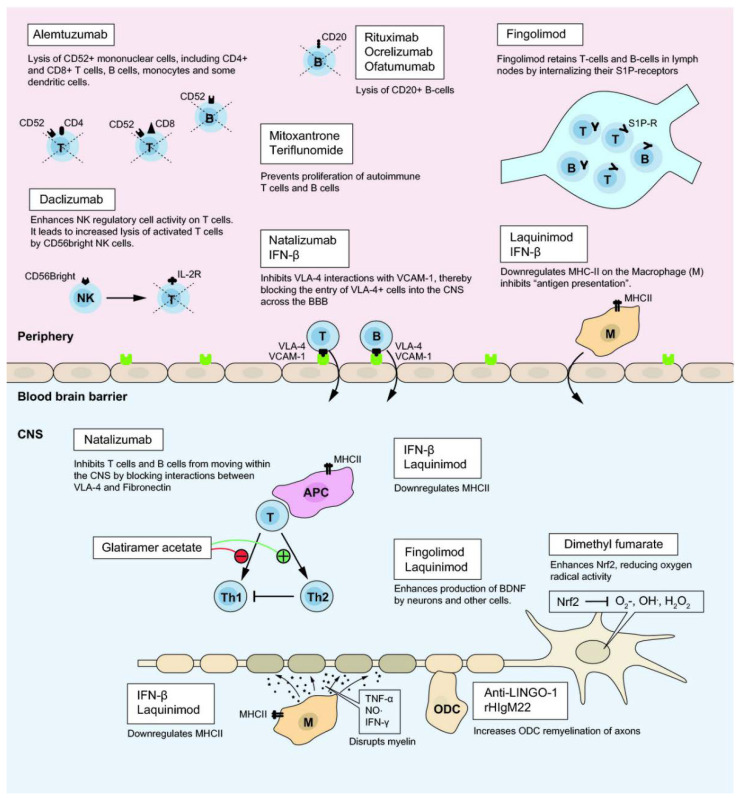
Suggested mechanism of action of several disease-modifying therapies (DMTs) (with the permission of [47]).

Current treatments for MS can be categorized into long-term immunosuppressant drugs, which have significant risks for various infections and cancer, and disease-modifying therapies (DMTs) designed to alter the progress of the disease via interference with B and T cells activity, and reduction of BBB disruption. For example, the more recently engineered monoclonal antibodies (mAbs) act via blocking α4 integrin interactions (e.g., natalizumab) or lysing immune cells exhibiting surface markers like CD20 (ocrelizumab, ofatumumab) [41] or CD52 (alemtuzumab). Due to their different mechanisms of action (Figure 3), DMTs’ efficacy and safety profiles [42] vary significantly. Presently, there exist more than 10 FDA (U.S. Food and Drug Administration) approved DMTs for RRMS aiming to reduce relapse level and severity of inflammation in CNS. DMTs can be classified based on the administration route as intravenous, self-injectable and oral formulations (Table 1) [16,23,31,43,44,45,46,47,48,49].

Among the FDA-approved DMTs, ocrelizumab, alemtuzumab and natalizumab seem to have the highest anti-inflammatory effect and to efficiently reduce relapses as proven by MRI scans [2,50]. Another approach for the treatment of MS involves the use of low-dose interleukin 2 (IL-2). This treatment is based on the weak in vivo response of effector T cells to low-dose IL-2 compared with Foxp3+ Treg cells which proliferate due to the expression of the high-affinity IL-2 receptor (CD25). This treatment has been shown to be well tolerated but, since non-specific expansion of the Foxp3+ Treg population cannot be excluded, it may effect susceptibility to infections and malignancies in some patients [51]. Interestingly, it has been shown that the more aggressive and less selective targeting of immune cells leads to more effective disease suppression, though at the cost of enhanced risk of side effects like infections and neoplasms due to decreased normal immune surveillance [27].

Despite the noteworthy advancements in the treatment of MS, the observed rates of progressive disability as well as of early mortality are still bothersome. Accordingly, there exists a need for safer, well tolerated and highly efficient treatments. This need is even higher for therapies capable of stopping or slowing the progression, and improving the disability in progressive MS [14,16,52,53,54]. Till now, only one therapy (ocrelizumab) appeared to be beneficial for the treatment of PPMS [14,16]. 

### 3.2. Antigen-Specific Immunotherapies

The Holy Grail for the treatment of MS is to specifically suppress the disease while at the same time allow the immune system to be functionally active against infectious diseases and malignancy. This could be achieved via the development of immunotherapies designed to specifically suppress immune responses to self-antigens [43,51,58,59,60]. Even though the detailed mechanisms of MS induction have not been fully clarified, a dominant hypothesis is that the loss of immune tolerance to myelin proteins like myelin basic protein (MBP), proteolipid protein (PLP) and myelin oligodendrocyte glycoprotein (MOG) leads to the recruitment of myelin-specific CD4+ T cells, resulting in myelin damage [14,61].

**Figure 4:**
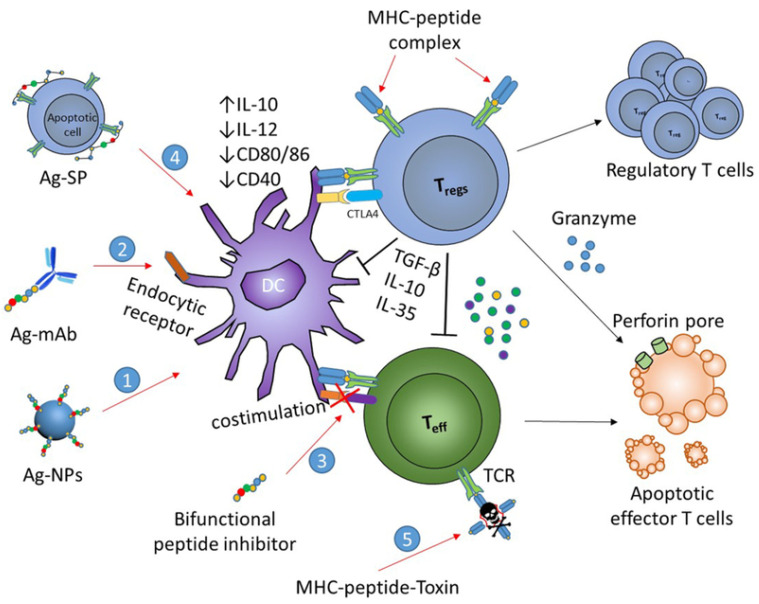
Bioconjugate-based approaches for the induction of Ag-specific tolerance in autoimmune diseases. The engineered bioconjugates target autoantigens and tolerogenic molecules to DCs (1); to facilitate antigen-processing via endocytic receptors (2); to hinder costimulation (3); to link to apoptotic cells for tolerogenic presentation (4); and to deliver toxin to autoantigen-specific T cells (5). These strategic approaches lead to peripheral tolerance as a consequence of anergy and deletion of cognate T cells, and/or induction of Tregs (with permission of [62]).

Antigen-specific immunotherapies are based on the introduction of self-antigens to APCs in the absence or presence of very low levels of costimulatory molecules (i) acting directly via TCR on effector T cells resulting in immunological anergy and deletion of pathogenic T cell clones (passive tolerance), and (ii) through activation, expansion, and differentiation of antigen-specific regulatory T cells which secrete anti-inflammatory cytokines (active tolerance) [62,63] (Figure 4). 

More specifically, an immunological synapse is established between APCs and T cells that is based on the formation of a trimolecular complex (signal 1) comprising the HLA class II molecule on the APC, the antigen (e.g., immunodominant epitope of a myelin protein) bound to this molecule and the TCR [64,65]. The establishment of the immunological synapse is the most vital process for the activation of effector T cells. In the absence of costimulatory molecules (signal 2), T cells become unresponsive to the antigen stimulation, a state known as anergy [65,66]. The presence of a costimulatory molecule exhibiting inhibitory properties could result to clonal deletion via apoptosis of the T cells. Autoreactivity of T lymphocytes can be also suppressed by the induction of regulatory T cells resulting in stable and long-term immune tolerance [59,65]. In vivo experiments have revealed that antigen-specific regulatory T cells are more effective than polyclonal Tregs regarding the control of organ-specific autoimmune diseases [67]. Finally, immune tolerance can be achieved via cytokine induced immune deviation, i.e., skewing of effector T cell subsets from Th1 and Th17 (proinflammatory phenotype) towards Th2 and Tr1 (anti-inflammatory phenotype) [59,65].

Antigen-specific therapies can be categorized according to the nature of the tolerogen (e.g., peptides derived from MBP, PLP, or MOG, mixtures of myelin derived peptides; altered peptide ligands; plasmids encoding myelin derived peptides, peptides related to TCR regions, attenuated myelin-specific T cells, tolerogenic DCs, antigen-coupled cells), the administration route (e.g., intravenous, subcutaneous, intraperitoneal, mucosal, epicutaneous, infusion of Ag-coupled cells) [14,43,51,59,65] and the antigen dose [68]. Since, antigen-specific therapies are thought to combine maximal efficiency with minimal side effects, they could be considered especially appealing [14]. On the other hand, they need to overcome major challenges in order to be efficiently used for the treatment of MS. 

The first challenge is that the target antigens in MS are not known and remain to be identified [14,27,65]. The disease is largely heterogeneous. It involves multiple autoantigens (contrary for example to neuromyelitis optica that involves reactivity to Aquaporin-4, AQP4) that can vary between patients depending on genetic characteristics, age, environmental and/or triggering factors, and duration of the disease [2,27,69,70]. It has been assumed that myelin targets like MBP, PLP and MOG are relevant, but this is mainly based on EAE models and not on MS patients. Furthermore, therapeutic efficiency in EAE cannot always be translated in MS. Accordingly, the interpretation of the above remains a crucial challenge for the translation of antigen-specific therapies from bench to bedside [27].

Furthermore, it should be noted that the clinical/neuropathological features of MS change noticeably with time [5,70]. Thus, not all patients will necessarily have similar responses to myelin antigen-specific immunotherapies [5]. Additionally, in chronic MS, the pattern of recognized autoantigens progressively increases during the course of the disease, due to a spread of the adaptive immunity to related self-antigens, a phenomenon recognized as epitope spreading [69,70]. Epitope spreading has been defined as the broadening of epitope specificity from the initial immunodominant epitope-specific immune response to other subdominant protein epitopes [71]. Epitope spreading can be categorized as “intra-molecular” related to shifting of immune responses between different epitopes of the same protein (e.g., MBP) and “intermolecular” related to the shifting of immune responses between two proteins (e.g., MBP and PLP) [27,72]. The hierarchy of immunodominant and cryptic epitopes is supposed to be dependent on a combination of peptide processing and presentation by various APCs, and also on the availability of epitope-specific T lymphocytes, taking into account the mechanisms of central and peripheral tolerance [71]. Accordingly, identifying the autoantigens that should be included in the therapeutic formulation can be rather challenging. This problem might be partially overcome via tolerance spreading, i.e., a gradual spread of the tolerance to the administered autoantigens also to other self-antigens which are involved in autoimmunity [70]. Elucidation of the cellular and molecular mechanisms involved in epitope spreading in MS is very important in order to design efficient antigen-specific immunotherapies for MS patients [71]. In this respect, therapeutic strategies targeting a broader array of epitopes may need to be pursued. Furthermore, since immune reactivity broadens with disease duration, antigen-specific immunotherapies should ideally be delivered early in the course of the disease when epitope spreading has not yet occurred, according to an optimized dosage and frequency schedule [14,27,65,73]. An alternative approach could be to achieve bystander suppression (i.e., modulation of the responses to one target antigen leads to modulation of the responses to neighboring target antigens). However, limiting evidence exists for such therapies [27].

Finally, another challenge regarding the translation of antigen-specific immunotherapies from bench to bedside is that the administration of tolerogenic vaccines to MS patients with inapparent infections could be immunogenic and worsen the course of the disease due to its presentation in the immune system in a pro-inflammatory environment. This has been the case in clinical trials with APL [74]. Thus, a crucial test for tolerogenic vaccines could be the in vivo assessment of their delivery in a proinflammatory environment, either after EAE onset, or by co-delivery of adjuvants and/or pro-inflammatory stimuli during EAE immunization [63].

Continuing research efforts towards the development of effective and safe antigen-specific therapies for MS gave rise to the epicutaneous administration of antigens (e.g., dermal patch loaded with myelin derived peptides) for the establishment of skin-induced immune tolerance in MS. The ability of skin DCs to induce myelin-specific tolerance has already been demonstrated in both in vivo experiments (Table 2) and early clinical trials [28,58]. Finally, oral tolerance has appeared to be efficient regarding the prevention of EAE, but significantly less efficient concerning the therapy of ongoing EAE and MS [75].

## 4. In Vivo Assessment of Tolerance-Inducing Vaccination in MS

### 4.1. Animal Model of MS

The typically used animal model of MS is that of the experimental autoimmune encephalomyelitis (EAE) [3,4,18,76,77,78,79,80]. EAE is an acute or chronic neuro-inflammatory brain and spinal cord disease [18] which can be induced in various animal strains such as mice, rats, guinea pigs, rabbits, and even primates [7], via immunization with spinal cord homogenate or with various myelin proteins (e.g., MBP, PLP, MOG) emulsified in complete Freund’s adjuvant (active EAE) [7,78,81]. EAE can be also transferred to naïve mice via adoptive transfer of T cells specific for myelin [8,78]. In EAE, myelin peptides are presented on MHC class II molecules to autoreactive T cells, together with costimulatory molecules (e.g., CD80 and CD86), resulting in activation of the T lymphocytes and, consequently, in an autoimmune attack on the myelin sheath [79]. EAE is principally mediated by myelin specific CD4+ T cells [20,78,82,83]. The clinical course of EAE varies based on the immunized animal species and the encephalitogenic antigen used for the inoculation. Usually the animals experience either an acute monophasic, progressive or not, disease, or a chronic relapsing-remitting disease. Ataxia, weight loss, sagging hind limb and paralysis are among the typical clinical signs of EAE [78]. Interestingly, various effective RRMS therapies (e.g., anti-inflammatory, immunomodulatory therapies) have been developed with the aid of EAE models. However, to date, no EAE model exists, that is capable of reproducing the specific features (e.g., clinical and neuropathological) of progressive MS. Therefore, despite the undeniable value of EAE for basic research concerning the mechanisms of brain inflammation and immune mediated CNS tissue damage, its value as model for MS is limited [18].

### 4.2. Myelin Peptide-Based Vaccination

#### 4.2.1. Immunodominant Myelin Petides

Myelin is a multilaminar sheath around nerve fibers comprising lipid bilayers and different proteins. The major myelin proteins are MBP and PLP which represent more than 75% of the total myelin protein. Additionally, myelin contains MOG [84] representing ~0.05% of the myelin proteins [7], myelin-associated oligodendrocyte basic protein (MOBP), oligodendrocyte-specific protein (OSP), myelin-associated glycoprotein (MAG), and Nogo-A [85].

While the etiology of MS is not clear yet, a favored hypothesis supported by experimental evidence indicates that the cross-reactive immune response between myelin derived epitopic peptides and viral or bacterial components can be considered as an important factor that contributes to the development of autoimmune T cells which initiate a demyelinating inflammatory response. Thus, the determination of the main epitopes of the encephalitogenic myelin and/or neuronal proteins that are implicated in MS is considered of major significance both for the development of antigen-specific therapies for MS and the elucidation of MS pathophysiology and etiology [85].

In recent decades, extensive studies have been performed aiming to identify the immunodominant epitopes recognized by T lymphocytes in MS. These studies have revealed that only the myelin proteins MBP, PLP, MOG, MOBP, and OSP can induce clinical EAE in laboratory animals and that autoimmune T cells against these proteins can be detected in MS patients. Other myelin proteins, like MAG and Nogo-A have been also identified as encephalitogenic proteins. Finally, some neuronal components (e.g., β-Synuclein, Neurofilament) have been found to exhibit encephalitogenic potential [85]. Antigen recognition takes place in the setting of a trimolecular complex formed by HLA, myelin peptide and TCR [64,86,87]. The immunodominant PLP epitopes which can be processed by human APCs lie within the PLP regions 30–60 and 180–230. Similarly, the PLP epitopes that activate T lymphocytes in EAE are within the 40–70, 90–120 and 180–230 regions of the protein [5]. Immunodominant epitopes of MOG that are recognized by encephalitogenic T cells in MS as foreign antigens are MOG_1–22_, MOG_35–55_ and MOG_92-106_ with the 35–55 epitope being the major immunodominant region of MOG [86]. Analysis of T-cell responses to MOBP in SJL/J mice indicated MOBP_15-36_ as the main encephalitogenic epitope of MOBP [85].

A cyclic analogue of MBP_87–99_ has been designed by Matsoukas and coworkers taking into consideration HLA (His^88^, Phe^90^, Ile^93^) and T-cell (Phe^89^, Lys^91^, Pro^96^) contact side-chain information. cyclo(87-99)MBP_87–99_ was shown to induce EAE, bind HLA-DR4, and enhance CD4+ T-cell proliferation, similarly to the linear MBP_87–99_ peptide [83]. Additionally, peptide analogues derived from the encephalitogenic peptide MBP_82–98_, the altered peptide ligand MBP_82–98_ (Ala^91^) and their cyclic analogues were synthesized by Deraos and coworkers and assessed regarding their binding to HLA-DR2 and HLA-DR4 alleles involved in the presentation of myelin epitopes to T cells. The cyclic MBP_82–98_ was shown to bind strongly to HLA-DR2 and to have a lower affinity to the HLA-DR4 allele. Both the cyclic and APL analogues of MBP_82–98_ were found to be promising and were selected to be further evaluated regarding their ability to modulate the responses of autoreactive T cells in MS [88]. In addition to the abovementioned studies, Tapeinou and coworkers developed a peptide compound comprising the MBP_85–99_ immunodominant epitope coupled to an anthraquinone derivative (AQ) via a disulfide (S-S) and six amino hexanoic acid (Ahx) residues. AQ-S-S-(Ahx)6MBP_85–99_ was found to bind reasonably to HLA II DRB1*-1501 antigen indicating the possibility of eliminating encephalitogenic T lymphocytes through generation of a toxic, thiol-containing moiety (AQ-SH) [89].

Yannakakis and coworkers used molecular dynamic simulations to study the interactions of the MOG epitope MOG_35–55_ with the HLA and TCR receptors during the formation of the trimolecular complex TCR-hMOG_35–55_-HLA DR2 [64]. They also used robust computational methods (e.g., molecular dynamics, pharmacophore modeling, molecular docking) to rationally design non-peptide mimetic molecules capable of binding with enhanced affinity to the T-cell receptor and not to the MHC-peptide complex, thus impeding the formation of the trimolecular complex [90].

To date various studies have assessed different myelin epitopes, as single peptides or mixtures of them, regarding their ability to induce antigen-specific tolerance in EAE animal models (Table 2).

#### 4.2.2. Altered Peptide Ligands (APLs)

Altered peptide analogues (APLs) of the immunodominant myelin protein epitopes have been successfully synthesized and applied in antigen-specific immunotherapies in vivo (Table 2). They are molecules where one or more amino acids in the sequence of the native immunodominant peptides, crucial for the interaction with the TCR, have been substituted. Depending on the substitutions, APLs can induce protective or therapeutic immune responses against EAE [91]. APLs can change agonist peptides into antagonist ones. Antagonistic peptides participating in the trimolecular complex MHC-peptide-TCR and causing suppression of EAE exhibit loss of their side chain interactions with the complementarity determining region 3 (CDR3) loop of the TCR. Substitution of large side chains interacting with the TCR with small side chain amino acids (e.g., Ala) causes antagonism and, therefore, inhibition of EAE symptoms. Moreover, APLs can switch Th1 cell response towards Th2 thus leading to disease suppression. Finally, APLs might activate regulatory T cells capable of antagonizing the deleterious actions of encephalitogenic cells in the CNS [83,87]. Accordingly, mutant cyclic peptides of MBP87-99 (e.g., cyclo(91-99)[Ala96]MBP87-99 and cyclo(87-99)[Arg91Ala96]MBP87-99) were shown to suppress the proliferation of a CD4 T-cell line from a MS patient, bind to HLA-DR4 and exhibit an increased Th2/Th1 cytokine ratio in peripheral BMCs derived from MS patients [83].

Molecular dynamics were applied by Mantzourani and coworkers to study the interactions of the MBP_87–99_ epitope and its antagonistic APLs (e.g., [Arg^91^, Ala^96^] MBP_87-99_ and [Ala^91,96^] MBP_87–99_) with the receptor HLA-DR2b [92]. 

#### 4.2.3. Y-MSPc

Kaushansky and coworkers [93,94] pursued a “multi-epitope-targeting” approach aiming to simultaneously neutralize T lymphocytes reactive against various major encephalitogenic epitopes. In this respect, they designed a recombinant synthetic protein comprising multiple epitopes of the human myelin protein (Y-MSPc). Y-MSPc was shown to efficiently inhibit the development of EAE induced in mice by a single epitope of myelin protein (classical EAE) or by a cocktail of five different encephalitogenic peptides (complex EAE) and suppress its progression, outperforming the single disease-specific epitope and the.mixture of peptides (Table 2).

#### 4.2.4. Cytokine-Neuroantigen (NAg) Fusion Proteins

**Figure 5:**
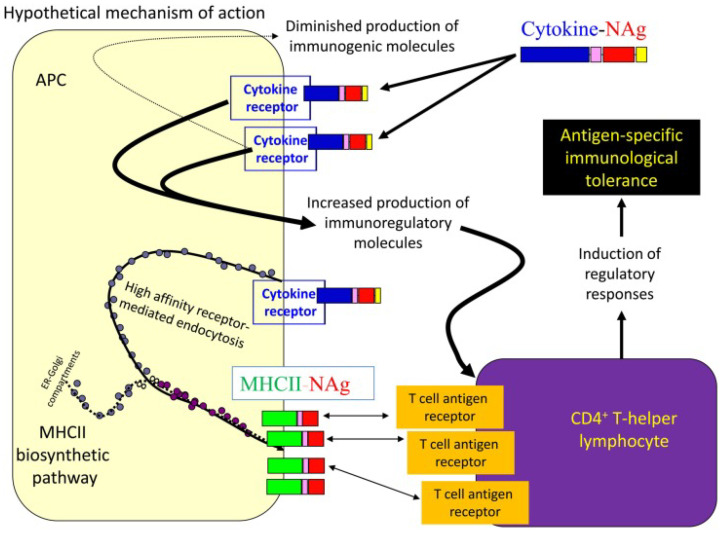
Mechanism of action of cytokine-NAg fusion proteins [96].

Fusion proteins consisting of a cytokine (N-terminal domain) fused with or without an appropriate linker to a neuroantigen (C-terminal domain) represent an emerging platform for antigen-specific vaccination [95,96]. Regarding their mechanism of action, the cytokine domain of the vaccine exhibits high affinity binding to specific surface cytokine receptors on certain subsets of APCs. This results in highly efficient uptake of the neuroantigen domain by these APCs, and its processing and presentation on MHC class II molecules to NAg-specific T lymphocytes. NAg tolerogenic presentation is assumed to induce regulatory responses and results in the establishment of antigen-specific immunological tolerance (Figure 5) [96,97].

Various single-chain cytokine-neuroantigen (NAg) fusion proteins (e.g., granulocyte-macrophage colony-stimulating factor (GMCSF)-NAg, IFNβ-NAg, IL16-NAg, IL2-NAg), where NAg comprises self-myelin epitopes, have been examined as potential tolerogenic and/or therapeutic antigen-specific vaccines in EAE mouse models (Table 2). The developed fusion proteins have been found to target APCs and to effectively prevent the induction of EAE when administered prophylactically as well as to suppress pre-developed EAE. Due to their combined preventive and therapeutic activities, the cytokine-NAg vaccines were characterized as both tolerogenic and therapeutic.The ranking order with respect to their inhibitory activity was the following: GMCSF-NAg, IFNβ -NAg > NAgIL16 > IL2-NAg > MCSF-NAg, IL4-NAg, IL-13-NAg, IL1RA-NAg. [96].

Apart from the aforementioned cytokine-NAg fusion proteins, the macrophage colony stimulating factor (MCSF)-NAg fusion protein was used in order to increase the presentation of NAg by macrophages. However, it was found to be less tolerogenic than GMCSF-Nag, thus indicating the latter fusion protein as the most suitable for antigen-specific vaccination [95,98]. Additionally, it was revealed that GMCSF-MOG does not require a non-inflammatory quiescent environment to effectively prevent the development of EAE which contradicts the previous knowledge regarding tolerogenic vaccines [95,98]. 

#### 4.2.5. Antibodies Coupled with Myelin Peptides

The dendritic and epithelial cell receptor with molecular weight equal to 205 kDa (DEC205) is expressed by DCs and enables antigen presentation. Injection of antigens (Ags) coupled to antibodies (Abs) specific for DEC205 into mice, at a low dose (e.g., ≤ 0.1 μg of fusion mAb [99]) leads to Ag presentation by nonactivated DCs, resulting in induction of regulatory T lymphocytes. In this respect, fusion of αDEC-205 Abs with MOG_35–55_ [100] and PLP_139–151_ [101] ameliorated EAE in mice. Similarly, Ring and coworkers synthesized single chain fragment variables (scFv) specific for DEC205. scFvs were subsequently fused with MOG (scFvDEC:MOG) and administered to mice both before and after induction of EAE. Significant prevention of EAE was observed by vaccination with scFv DEC:MOG before immunization. In addition, administration of scFv DEC:MOG post immunization led to substantial alleviation of the clinical symptoms of the disease [102]. On the other hand, Tabansky and coworkers targeted the dendritic cell inhibitory receptor 2 (DCIR2) receptor with αDCIR2 Abs fused to PLP_139–151_ and observed significant alleviation of EAE clinical symptoms [79]. In another approach, Kasagy and co-workers demonstrated that administration of anti-CD4 and anti-CD8 Abs followed by injection of PLP_139–151_ resulted in substantially lower EAE scores and reduced rate of relapses in chronic disease in mice [103] (Table 2). 

#### 4.2.6. Recombinant T-cell Receptor Ligands (RTLs)

Antigen-specific immunosuppression can be induced via the utilization of MHC-peptide complexes as specific TCR ligands interacting with autoimmune T cells in the absence of co-stimulatory molecules. A recombinant TCR ligand (RTL) typically comprises a single polypeptide chain encoding the β1 and α1 domains of MHC class II molecules linked to a self-antigen [104] and represents the minimal interactive surface with antigen-specific TCR. RTLs fold in a similar manner to native four-domain MHC/peptide complexes but they deliver qualitatively different, suboptimal signals which cause a “cytokine change” to anti-inflammatory factors in targeted autoreactive T cells. Treatment with RTLs could reverse the clinical/histological signs of EAE in different experimental cases (e.g., MBP-induced monophasic disease, MOG peptide-induced chronic EAE, PLP-induced relapsing remitting EAE) and even promote recovery of myelin and axons in mice with chronic disease [105,106,107] (Table 2).

Alternatively, RTLs could involve natural or recombinant *α*_1_*α*_2_ and *β*_1_*β*_2_ MHC class II domains covalently or noncovalently linked with encephalitogenic or other pathogenic peptides. These specific RTLs could bind both to the TCR and the CD4 molecule on the T cells surface via the β_2_ MHC domain and were shown to hinder the activation of T cell and thus prevent EAE in rodents [108].

#### 4.2.7. Bifunctional Peptide Inhibitors (BPIs)

**Figure 6:**
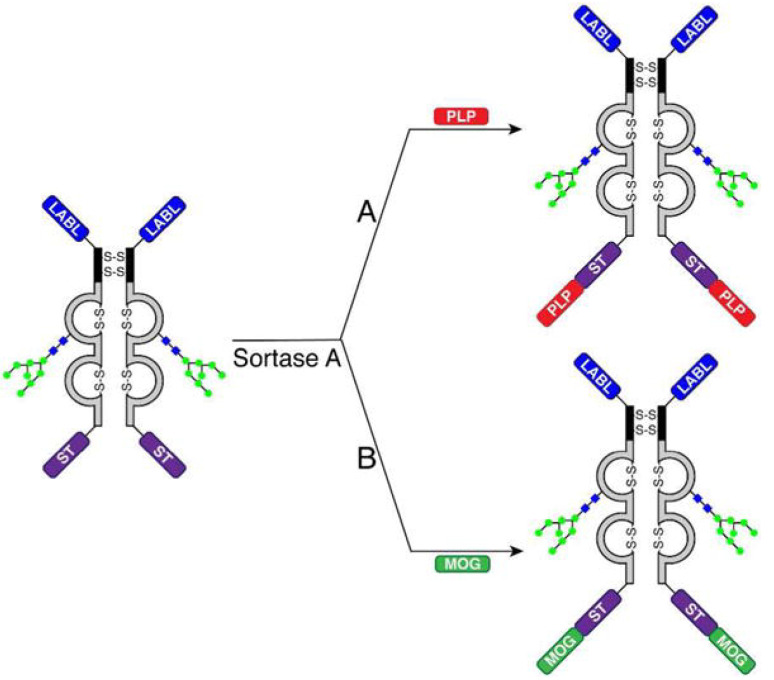
Sortase-mediated addition of two different antigens (A) PLP and (B) MOG to the C-terminus of LABL-Fc-ST (with permission of [109]).

Bifunctional peptide inhibitors (BPIs) are a promising novel class of peptide conjugates which are designed to selectively impede the maturation of myelin specific T cells. They comprise an immunodominant myelin protein epitope tethered to a signal-2-blocking peptide derived from lymphocyte function-associated antigen-1, LFA-1 (i.e., a T cell protein binding to intercellular adhesion molecule-1, ICAM-1) [109] (Figure 6). It is hypothesized that they bind at the same time to MHC-II and ICAM-1 on APCs thus inhibiting the immunological synapse formation during APC and T cell interactions [110]. The development of molecules that could target more than one epitope is crucial for the application of BPI technology in MS [111]. The performance of BPIs with respect to the induction antigen-specific immune tolerance has been studied in EAE animal models (Table 2).

#### 4.2.8. Antigen-Drug Conjugates

Antigen drug conjugates (AgDCs) combine two therapeutic approaches (e.g., antigen-specific immunotherapies and immunomodulatory agents) to treat autoimmune diseases. Via chemical conjugation, the Ag could target the immunomodulatory agent to diseased cells thus minimizing side effects. AgDCs are assumed to exhibit increased affinity specificity through targeting cognate B cell receptors or endogenous autoantibodies. AgDCs formation entails the selection of an appropriate pair of antigen and immune modulator, and a linking scheme. An AgDC combing PLP_139–151_ and dexamethasone (PLP_139−151_-DEX) was administered to mice induced with EAE. It was shown that the AgDC protected the mice from developing clinical symptoms during the 25-day study [61] (Table 2).

### 4.3. DNA Vaccination

**Figure 7:**
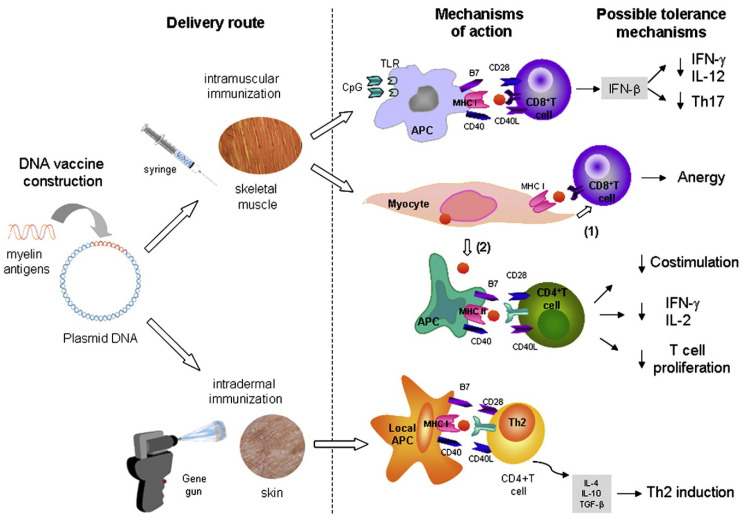
Mechanisms of immune tolerance induction by DNA plasmid vaccines (with permission of [139]).

Deoxyribonucleic acid (DNA) vaccination is considered a promising antigen-specific approach for the treatment of MS [91,136,137,138]. DNA plasmid vaccines for tolerance induction in MS comprise a bacterial plasmid encoding myelin antigen(s). Expression is controlled by a mammalian promoter and a transcription terminator. They are administered either as naked DNA or with the aid of carriers (e.g., cationic lipids, cationic liposomes, polymeric particles), via the intramuscular or intradermal (e.g., “gene gun” delivering gold particles coated with pDNA vaccines) administration routes. Vaccination leads to DNA uptake and gene expression by the cells at the injection site [139,140]. Induction of immune tolerance is achieved via the following potential mechanisms (Figure 7). After intramuscular injection, myocytes are the main transfected cells, as well as few APCs. Antigens are then presented by the following mechanisms: i) myocytes process and present the antigen to T cells leading to T cell anergy ii) myocytes produce and secrete antigen that is taken up by APCs, which subsequently activate T cells. This results in loss of T cell co-stimulation through CD28, downregulation of IL-2, production of IFN-γ and reduced T cell proliferation. Intramuscular injection can also induce IFN-β via TLR9 activation due to the presence of CpG in the plasmid backbone [140], leading to downregulation of IL-12, IFN-γ, and Th17 cell responses. Following intradermal administration, DNA is delivered directly into the resident APCs (e.g., Langerhans and dermal cells). Intradermal vaccination leads to the secretion of regulatory cytokines (e.g., IL-4, IL-10, and TGF-β) thus resulting in the induction of anti-inflammatory Th2 immune responses [139,141]. Balance between tolerance induction and inflammatory immune response can be controlled by the administration route, antigen dose, and modification of the DNA-encoded antigen [141]. Numerous data from in vivo studies with the EAE animal model (Table 3), have demonstrated the efficiency of DNA plasmid vaccines at inhibiting MS via inducing T regulatory cells or anergy, clonal deletion, and immune deviation [139].

### 4.4. Cell-Based Vaccination

#### 4.4.1. Antigen-Specific Tolerogenic Dendritic Cells (tolDCs)

**Figure 8:**
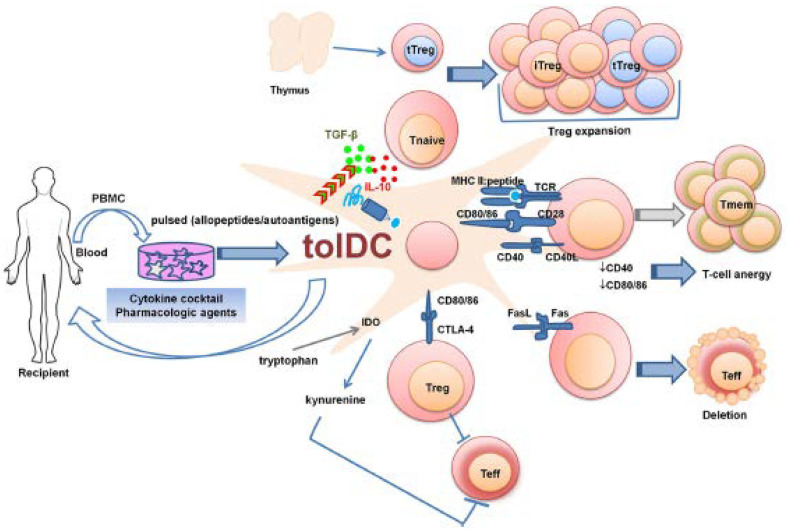
Strategies to generate tolDCs for clinical therapeutics [153].

Dendritic cells (DCs) have a critical role in initiating adaptive immune responses in order to eliminate invading pathogens as well as in inducing tolerance towards innocuous components so as to maintain immune homeostasis [149]. Tolerogenic dendritic cells (TolDCs) are considered an attractive therapeutic approach for the induction of antigen-specific tolerance in autoimmune diseases [150,151]. To date various protocols have been developed for the in vitro generation of clinical-grade tolerogenic DCs ([35, 152] (Figure 8) [153]) for antigen-specific immunotherapies. Autologous peripheral blood mononuclear cells (PBMCs) or bone marrow derived cells (BMDCs) are differentiated into tolDCs by numerous pharmacologic agents (e.g., immunosuppressive drugs such as rapamycin, cytotoxic T-lymphocyte-associated protein 4 (CTLA-4) Ig, corticosteroids; cyclic AMP inducers such as prostaglandin E2 and histamine; chemicals like vitamin D3, aspirin, etc.; proteins and neuropeptides like HLA-G, vasoactive intestinal peptide, etc.) and immunomodulatory cytokines (e.g., IL-10, TGF and low doses of GM-CSF) [150,153] and are further pulsed in vitro with autoantigens, encephalitogenic peptides, apoptotic cells, etc. [153]. tolDCs can display an immature or a semi-mature phenotype which is characterized by altered cytokine production and low expression of MHC and co-stimulatory molecules [150]. 

Depending on the experimental protocol, the molecules used to induce tolerogenic properties, and the targeted cell population, tolDCs use different mechanisms of regulation to induce tolerance (Figure 8), including conversion to a regulatory T cell phenotype, induction of anergy, and antigen-specific deletion of T cell clones [19,35,150,152,153,154]. Lately, their ability to induce regulatory B cells secreting IL-10 has been also demonstrated [152]. TolDCs can be categorized into induced tolDCs (itDCs) (i.e., those acquiring their tolerogenic features in vitro or in vivo as described above and contribute to the maintenance of tolerance even under proinflammatory conditions) and natural tolDCs (ntDCs) (i.e., DCs present in the spleen and other lymphoid sites which inherently aid to establish tolerance in the absence of danger signals) [155].

**Figure 9:**
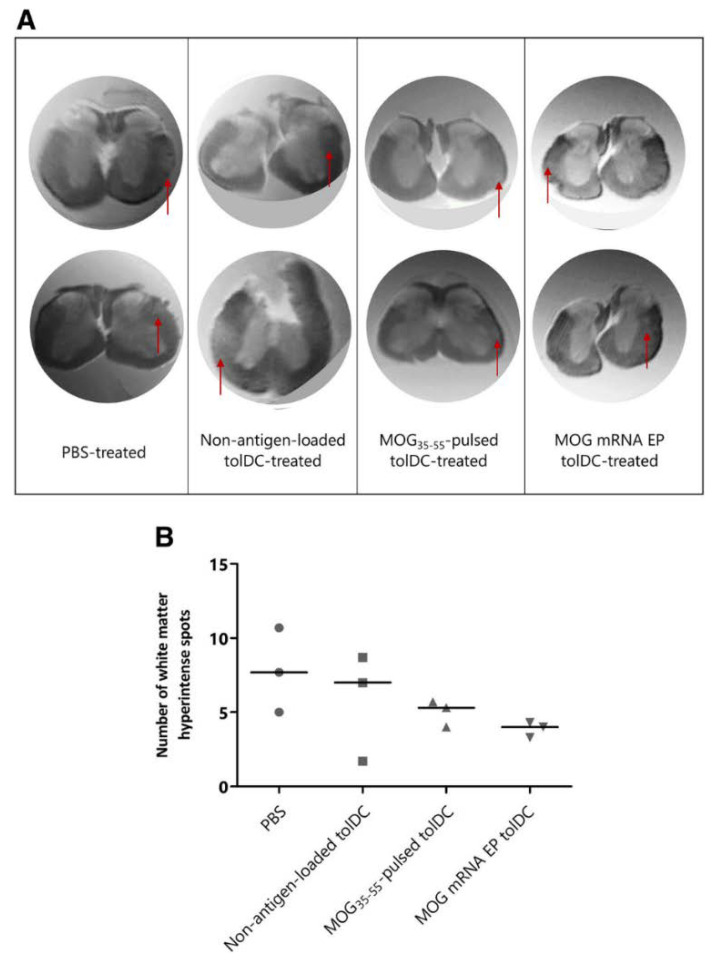
Evaluation of inflammatory lesion load within the spinal cord of tolDC-treated and PBS-treated mice using ex vivo MRI imaging. (**A**) Representative MRI of spinal cord with hyperintense white matter spots marked with a red arrow. Two representative axial slices are shown per treatment group. (**B**) The total number of hyperintense white matter spots along the entire spinal cord was quantified as a measure of lesion load in three mice per treatment group. Results are presented as individual scores for hyperintense spots with median [154].

The therapeutic potential of tolDCs has been demonstrated in the EAE model of MS (Table 4) (Figure 9). A key challenge is the translation of the in vivo results to humans. In this respect, it will be critical to correlate clinical efficiency with variation of immunological parameters and, accordingly, to define the best administration route and the effective dose of cells for this route [152]. Progress in the scientific areas of recombinant protein expression, genome editing and nanotechnology-based drug delivery systems, combined with improved immunization protocols, could further improve the promising tolDC vaccination in the furure [150].

#### 4.4.2. T Cell Vaccination (TCV)

**Figure 10:**
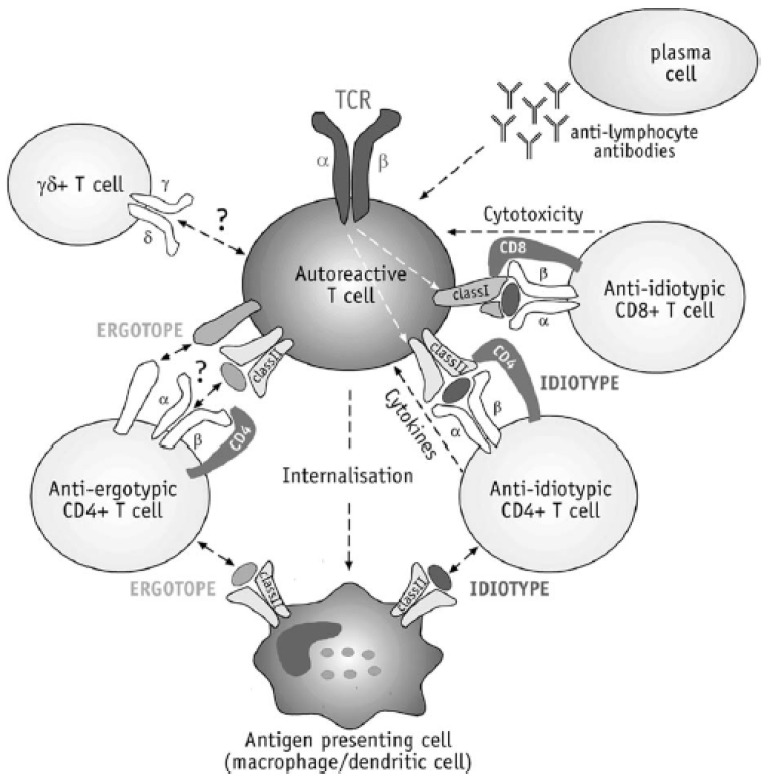
Complexity of anti-vaccine responses induced by TCV (with permission of [29]).

T cell vaccination involves the extraction of myelin reactive T cells from MS patients and their re-injection after irradiation in order to induce protective immunity [12,80,141,156]. To prepare T-cell vaccines, CSF mononuclear cells or blood PBMC’s are stimulated with myelin antigen, and are then expanded specifically for the selected myelin peptide till an adequate population of cloned T cells is available. The latter are activated with antigen, and attenuated via exposure to radiation (6–12,000 Rads) to avoid proliferation after injection [156,157]. In clinic, the TCV protocol also involves multi-epitope TCR peptides [80]. TCV has been found to specifically suppress autoreactive T cells in MS via induction of a complicated anti-ergotypic and anti-idiotypic regulatory network or T cell deletion [80,91,156]. Various typical cytokines and lymphocyte phenotype transfer have been shown to participate in the depletion of the autoreactive T cells and the reversion of abnormal autoimmune responses [80] (Figure 10).

#### 4.4.3. Antigen-Coupled Cells

Intact proteins (e.g., myelin proteins) as well as multiple peptides (e.g., MBP, PLP, and MOG derived peptides) can be coupled to a single cell (e.g., splenocyte [158,159], erythrocyte [67,160]) [86] (Table 4), thus permitting concurrent targeting of various T-cell specificities. This could be critical for antigen-specific immunotherapy in MS, where immune tolerance to multiple T-cell epitopes is considered necessary for the disease treatment due to epitope spreading. Contrary to protein/peptide-induced tolerance, vaccination with protein/peptide-coupled cells lowers the risk of anaphylaxis, since the antigen is chemically crosslinked to the cell surface. Vaccination with antigen-coupled cells has been found to prevent the active- and passive-transfer. Finally, tolerance induction with Ag-coupled cells can help define immunodominant myelin antigens, since the disease progression can be impeded by cells coupled with the spread epitope [75].

### 4.5. Carrier-Aided Vaccination

In recent decades, different strategies have been pursued for the development of carriers [175,176,177,178,179] loaded/conjugated with myelin antigens or combinations of myelin peptides and immunomodulating agents. The developed carriers have been designed to target TCR signaling pathways, as well as cytokines and co-signaling molecules, aiming to enhance TCR-mediated tolerance [30,62,177]. Various biomaterials (e.g., polymers, lipids) have been formulated into micro- or nanoparticles, self-assembled into different structures, or formed molecular conjugates with self-antigens (e.g., conjugation of self-antigens with polymers, antibodies, small molecules). Both nanoparticles (NPs) and microparticles (MPs) can be uptaken by APCs thus enhancing the intracellular delivery of myelin antigens and imunnomodulators [180,181].

#### 4.5.1. Polymer Particles

Polymer micro- and nanoparticles loaded with self-antigens and/or immunomodulatory molecules have recently emerged as ideal carriers for tolerogenic vaccines since their properties (e.g., particle size, composition, antigen/immunomodulator loading) can be fine-tuned to induce peripheral tolerance. Furthermore, NPs can be employed as platforms to regulate the doses and delivery times not only of the self-antigens but also of the tolerogenic adjuvants that are required to promote tolerance [70].

Poly(lactic-co-glycolic acid) (PLGA) NPs are non-toxic, biodegradable/biocompatible and have the advantage of being FDA approved for various clinical uses including drug delivery, diagnostics, etc. Additionally, surface functionalization strategies may improve their interaction with cells, thus optimizing cell targeting and vaccine performance. PLGA NPs are the most extensively assessed nanocarriers in pre-clinical models of autoimmune diseases and their effectiveness regarding antigen-specific immunotherapies (Table 5) represents a proof-of-concept of the feasibility of nanoparticle-aided tolerogenic vaccination. Furthermore, their successful application in animal models appears encouraging concerning potential translation to humans [70].

#### 4.5.2. Soluble Antigen Arrays

Soluble antigen arrays (SAgAs) are synthesized by co-grafting the immunodominant epitope PLP_139–151_ and LABL peptide (i.e., ligand of the intercellular adhesion molecule 1, ICAM-1) to hyaluronic acid (HA) via a hydrolysable oxime bond [182,183]. Their size can be fine-tuned to allow them to drain to the lymph nodes [183]. Another key factor affecting their drainage is the injection site and the molecular weight of HA. For example, following s.c. injection, HA can drain to the lymphatics and its retention time can be affected by its molecular weight [183]. 

**Figure 11:**
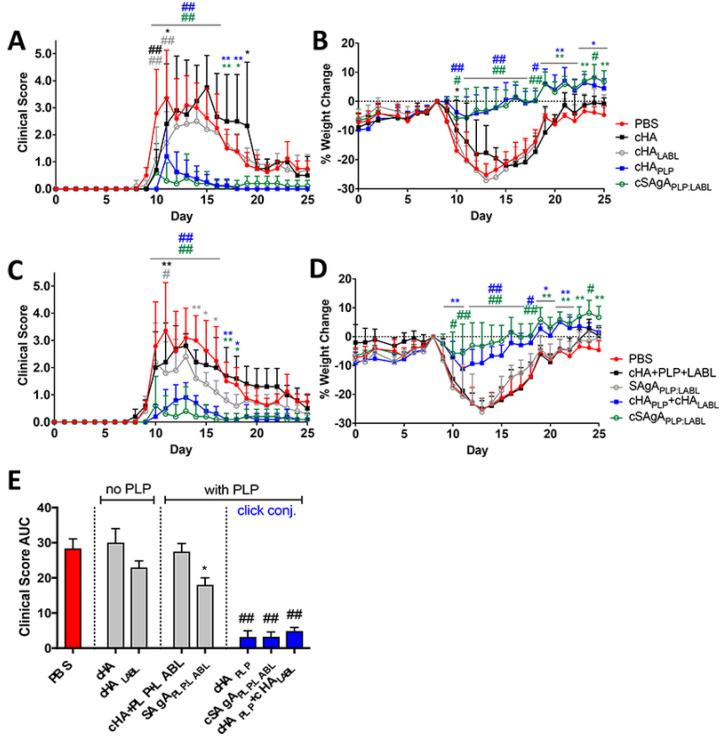
EAE in vivo response to click conjugates (cHA, cHALabl, cHAPLP, and cSAgAPLP:LABL) as measured by (**A**) clinical disease score and (**B**) percent weight loss. EAE in vivo response to groups containing both PLP and LABL (cHA+PLP+LABL, SAgAPLP:LABL, cHAPLP+cHALABL, and cSAgAPLP:LABL) as measured by (**C**) clinical disease score and (**D**) percent weight loss. Data represent mean ± SD (*n* = 5); statistical significance compared to PBS negative control was determined by two-way ANOVA. (**E**) Cumulative EAE in vivo response as measured by clinical disease score area under the curve (AUC) derived from subfigures A and C. Data represent mean ± SEM (*n* = 5); statistical significance compared to PBS negative control was determined by ordinary one-way ANOVA followed by Dunnett’s post hoc test. (* *p* < 0.05, ** *p* < 0.01, # *p* < 0.001, ## *p* < 0.0001, color coded according to group) (with permission of [185]).

The efficiency of the hydrolysable SAgA_PLP-LABL_ to suppress disease in mice with EAE has been reported in various studies (Table 5) and has been attributed to the simultaneous delivery of the myelin derived antigen and the cell adhesion signal [182]. Furthermore, earlier in vitro studies indicated that SAgAs demonstrate Ag-specific binding with B lymphocytes, target the B cell receptor (BCR) and reduce BCR-mediated signaling [184]. Based on the abovementioned experimental results indicating BCR engagement as the mechanism of action of SAgA_PLP-LABL_ Hartwell and coworkers developed a novel version of SAgA_PLP-LABL,_ the cSAgAPLP:LABL (click SAgA), employing non-hydrolysable conjugation chemistry (e.g., copper-catalyzed azide-alkyne aycloaddition) [184,185]. cSAgAPLP:LABL was found to significantly reduce or inhibit BCR-mediated signaling and to exhibit enhanced in vivo efficiency in comparison with the hydrolytically unstable SAgA_PLP-LABL_ [184,185] (Figure 11). 

#### 4.5.3. Immune Polyelectrolyte Multilayers (iPEMs)

It has been recently shown that excess signaling via inflammatory pathways such as toll-like receptors (TLRs) is involved in the pathogenesis of autoimmune diseases. Accordingly, the co-delivery of immunodominant myelin peptides with GpG oligonucleotide, a regulatory ligand of TLR9, could potentially limit TLR signaling during the differentiation of myelin-specific T lymphocytes, thus redirecting their differentiation towards a tolerogenic phenotype like the regulatory T cells. In this respect, immune polyelectrolyte multilayers (iPEMs) were formed using a layer-by-layer approach to co-assemble modified myelin peptides with GpG oligonucleotide. These nanostructures have key characteristics of biomaterial-based nanocarriers, such as tunable physicochemical properties and loading capacity, ability to deliver various active ingredients, etc., lacking, however, synthetic components that could exhibit inflammatory properties.

In in vitro studies, iPEMs have been shown to limit TLR9 signaling, decrease activation of DCs, and polarize myelin-specific T lymphocytes towards a tolerogenic phenotype. Additionally, they have been found to reduce inflammation and induce tolerance in mice with EAE [186,187] (Table 5).

#### 4.5.4. pMHC-Nanoparticles (pMHC-NPs)

The “two signal theory” states that two different signals are required for the activation of naive T cells: (i) engagement of the TCR with its cognate pMHC target, and (ii) a co-stimulatory signal from molecules selectively expressed on professional APCs’ surface. It is well known that engagement of the TCR on the surface of a naive T cell without co-stimulation results in the induction of apoptosis or anergy.

The development of pMHC-nanoparticles (pMHC-NPs) for the treatment of autoimmune diseases was based on the hypothesis that pMHC-coated NPs would diminish the responses of autoreactive T cells more efficiently compared with soluble pMHC complexes. This could be due to (i) their multimeric valency, (ii) their potentially superior TCR cross-linking properties compared with “artificial APCs”, and (iii) the protection of the NP-bound pMHC molecules from degradation [104]. The ability of pMHC-NPs to stop the progression of EAE was assessed with in vivo experiments in mice (Table 5).

#### 4.5.5. Mannan-Peptide Conjugates

**Figure 12:**
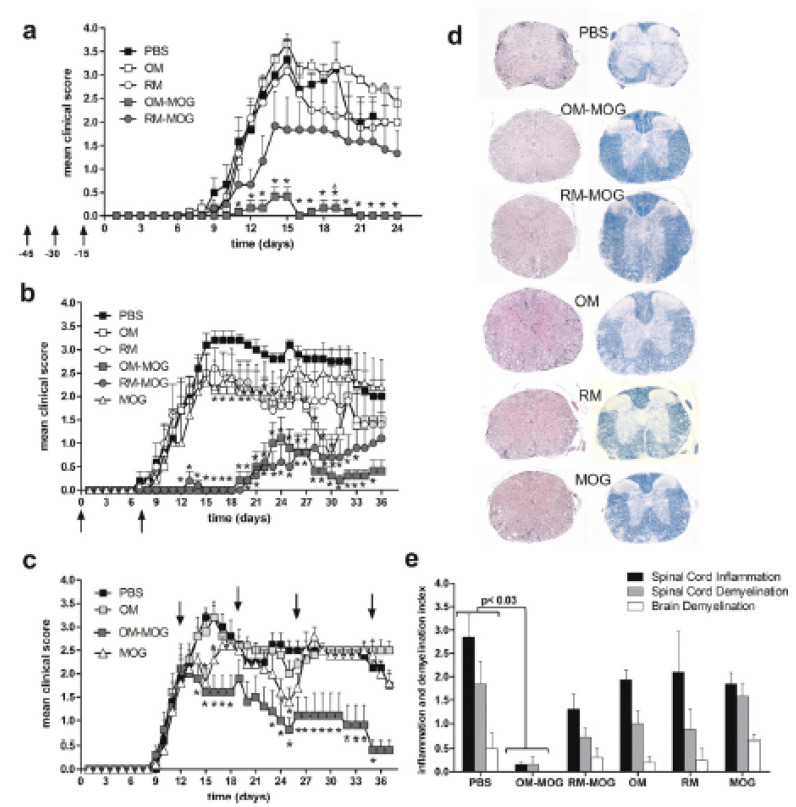
(**a**) Mean clinical scores of MOG-EAE in groups of mice vaccinated i.d. with OM-MOG, RM-MOG, OM, RM, or PBS at indicated time points (arrows) before immunization for EAE induction. (**b**) Mean clinical scores of MOG-EAE in groups of mice vaccinated i.d. at indicated time points (arrows) relative to immunization. (**c**) Mean clinical scores of MOG-EAE in groups of mice injected i.d. at indicated time points (arrows) after immunization. The results shown are from one representative of two (**b**,**c**) or three (**a**) independent experiments. (**d**,**e**) Vaccination with OM-MOG protects C57BL/6 mice from spinal cord inflammation and demyelination during MOG-EAE. (**d**) Inflammatory cell infiltration (left column) and demyelination (right column) were visualized on day 24 following immunization. (**e**) Quantification of spinal cord inflammation (black bars) and demyelination (grey bars) as well as brain demyelination (white bars) in all experimental groups. Representative data from five animals per group are shown. Statistical significance after comparisons between groups of mice (using the Kruskal-Wallis test) or histopathology indices (using Student’s *t* test) is shown (*, *p* < 0.05). Triangles (**a**) indicate time points where pair-wise comparison between OM-MOG and RM-MOG groups also show significant differences (with permission of [188]).

Based on previous studies with the yeast polysaccharide, mannan, Tseveleki and coworkers, examined mannan conjugation with immunodominant myelin epitopes as an approach to divert the differentiation of myelin-specific T lymphocytes towards a regulatory phenotype, thus decreasing the mice susceptibility to EAE. It was shown that the administration of the synthesized conjugates to mice in both prophylactic and therapeutic vaccination protocols resulted in the induction of antigen-specific T cell tolerance and significant amelioration of EAE clinical and histopathological symptoms. [188] (Figure 12) (Table 5). According to these results, it was speculated that conjugation of MOG epitopes to mannan may modulate the autoimmune response in humans, thus potentially reducing the symptoms of MS [188].

#### 4.5.6. Liposomes

Liposomes are tiny vesicles featuring an aqueous core surrounded by a lipid bilayer. They can encapsulate both hydrophilic and hydrophobic drugs and target them to specific cell surfaces via appropriate functionalization. Various types of liposomes have been already approved for clinical use (e.g., delivery of therapeutics, vaccination) and can be designed to induce or tolerate immune responses [189]. Pujol-Autonell and coworkers reported the beneficial effect of MOG peptide loaded liposomes in treating mice with EAE. Liposomes successfully delayed the onset, suppressed the severity and decreased the incidence of the disease [190]. Similarly, Belogurov and co-workers demonstrated that mannosylated liposomes containing MBP_46–62_ could significantly reduce EAE clinical signs in Dark Agouti (DA) rats [189]. Interestingly liposomes loaded with MBP_46–62_, MBP_124–139_, and MBP_147–170_ and targeting CD206 were proven to be safe and well-tolerated and to normalize cytokine levels in RRMS and SPMS patients [191,192].

#### 4.5.7. Microneedle Patches

Pires and coworkers proposed the use of minimally invasive microneedle patches for the delivery of myelin peptides, as an alternative therapeutic strategy for skin mediated antigen-specific immune tolerance in MS [178].

## 5. Clinical Trials

Various tolerance-inducing vaccination approaches (e.g., immunodominant myelin epitopes, APLs, DNA vaccination, attenuated autologous myelin reactive T cells, tolerogenic DCs, TCR peptide vaccination, nanocarriers loaded with encephalitogenic myelin peptides, etc.) with promising outcomes in experimental MS models have already reached the clinical development phase. Their safety, feasibility, and efficiency in inducing antigen-specific immune tolerance and reducing MRI-detected disease activity in patients with relapsing remitting and progressive MS have been preliminary demonstrated in phase I and II clinical trials [14,136,139] (Table 6).

## 6. Conclusions

Several exciting vaccination strategies targeting the induction of antigen-specific immune tolerance in MS have been developed during the last decades, based on a single epitope or cocktails of immunodominant epitopes of myelin proteins, altered peptide ligands, DNA vaccines, tolerogenic DCs pulsed with myelin peptides, attenuated autologous myelin reactive T cells, TCR peptide vaccines, conjugates of autoantigens with various types of cells, and different types of carriers (e.g., particles, vesicles, self-assembled structures, or molecular carriers) associated with myelin epitopes. Most of these approaches have demonstrated promising results in animal models of experimental autoimmune encephalomyelitis both in prophylactic and therapeutic vaccination protocols. They successfully prevented the disease or delayed the disease onset, reduced its clinical and pathological symptoms and decreased the number of relapses, or, in a therapeutic scheme, they reversed the clinical and histological signs of the disease. Accordingly, numerous of the abovementioned strategies reached the clinical development phase, and their safety, feasibility, and efficacy were assessed in both phase I and II clinical trials. However, the results from these trials have not indicated the same level of efficiency as in preclinical models. Even though different tolerance-inducing vaccination strategies were proven safe and well tolerated, and in some cases succeeded in inducing tolerogenic responses to patients, no major advances have been reported with respect to clinical efficiency. Consequently, despite the intensive research efforts, up to the present time, no FDA approved antigen-specific immunotherapy is available for treating MS patients. It appears that antigen-specific immunotherapies still face various major challenges such as the involvement of multiple autoantigens that can vary between patients, the epitope spreading, the vaccination of patients with inapparent infections, etc. These challenges need to be overcome in order to allow tolerogenic vaccines to play a major role in the treatment of MS patients. Progress in the scientific areas of recombinant protein expression, genome editing, and smartly designed carriers, combined with better understanding of MS immunopathogenesis and improved immunization protocols, could potentially improve these vaccination strategies in the future. Additionally, further clinical studies, such as phase II and III, including placebo groups, will be required in order to more realistically assess the clinical effectiveness of these interesting antigen-specific immunotherapies in both RRMS and SPMS patients.

## Figures and Tables

**Table 1:** Disease-modifying-therapies for RRMS (based on [16,23,43,45]).

Therapeutic Molecule	Commercial Name	Year of Approval	Admin. Route	Admin. Frequency	Mode of Action	Side Effects
IFN-β1a	Avonex^®^ Rebif^®^	1993	i.m. s.c.	Once a week Three times a week	Decrease of proinflammatory and increase of anti-inflammatory cytokines; decreased migration of inflammatory cells across the BBB; decrease of Th17 cells; modulation of T and B cells.	Symptoms similar to those of flu; leukopenia; liver damage.
pegIFN-β1a	Plegridy^®^		s.c.	Once per two weeks	Decrease of proinflammatory and increase of anti-inflammatory cytokines; decreased migration of inflammatory cells across the BBB; decrease of Th17 cells; modulation of T and B cells	Symptoms similar to those of flu; leukopenia; liver damage.
IFN-β1b	Betaseron^®^ Extavia^®^	1993	s.c.	Once per two days	Decrease of proinflammatory and increase of anti-inflammatory cytokines; decreased migration of inflammatory cells across the BBB; decrease of Th17 cells; modulation of T and B cells; down regulation of MHC expression on APCs.	Symptoms similar to those of flu; leukopenia; liver damage.
Glatiramer acetate	Copaxone^®^	1996	s.c.	-	Decrease of proinflammatory and increase of anti-inflammatory cytokines; decrease of Th17 cells; increase of Th2 cells and Tregs; blocking of pMHC.	Erythema; induration; heart palpitations; dyspnea; tightness of chest; flushes/anxiety.
Dimethyl fumarate	Tecfidera^®^	2013	oral	Twice or three times per day	Anti-inflammatory-Increase of Th2 cells; anti-oxidative stress; neuroprotection through activation of Nrf-2 pathway.	Flushes; vomit; diarrhea; nausea; decrease of WBC.
Teriflunomide	Aubagio^®^	2012	oral	Once per day	Inhibition of dihydroorotate dehydrogenase; inhibition of T and B cells;	Lymphopenia; nausea; hypertension; fatigue; headache; diarrhea; peripheral neuropathy; acute renal failure; alopecia.
Fingolimod	Glenya^®^	2010	oral	Once per day	S1P receptor modulator; preventing the circulation of lymphocytes in non-lymphoid tissues including the CNS.	Weakening of heart rate; hypertension; macular edema; increased liver enzymes; decreased lymphocyte levels.
Siponimod [55]	Mayzent^®^	2019	oral		Binding to S1P-1 and S1P-5	
Ozanimod [56]	Zeposia^®^	2020 USA	oral		S1P receptor agonist	
Laquinimod			Oral		Immunomodulation of T cells, DCs and monocytes; neuroprotection of astrocytes; decrease of proinflammatory and increase of anti-inflammatory cytokines; reduced infiltration of cells into the CNS.	No severe cardiac adverse effects were detected during Phase III clinical trials.
Cladribine [57]	Mavenclad^®^	2017 EU 2019 USA			Reduction of circulating T and B cells.	Risk of cancer
Mitoxantrone	Novatrone^®^	2000 USA	i.v.	Once per three months	Cytotoxic for B and T cells; reduction of Th1 cytokines; inhibition of type II topoisomerase.	Cardiotoxicity; leukemia
Methylprednisolone			i.v.	-	Immunosuppression; anti-inflammatory effects.	Risk of infections; retention of sodium; glucose intolerance; mood disturbances.
Dalfampridine	Ampyra^®^		oral	Twice per day	Blocking of potassium channel; improvement of motor symptoms.	
Natalizumab	Tysabr^®^	2004	i.v.	Once per 28 days	Targeting α4-integrin	Progressive multifocal leukoencephalopathy.
Ofatumumab	Arzerra^®^		i.v.	Once per two weeks	Targeting CD20	
Ocrelizumab	Ocrevus^®^		i.v.	Once per six months	Targeting CD20	
Alemtuzumab	Lemtrada^®^	2013 EU	i.v.	Once a year	Targeting CD52	High risk of infections Graves’ disease
Daclizumab	Zinbryta^®^		s.c.	Once per month	Targeting CD25	
Rituximab	Rituxan^®^		i.v.	-	Targeting CD20	Chills; nausea; hypotension
Obinutuzumab	Gazyva^®^		i.v.	-	Direct cell death	Risk of infections; nausea; thrombocytopenia; neutropenia

IFN: interferon; i.m.: intramuscular; s.c.: subcutaneous; BBB: blood-brain barrier; MHC: major histocompatibility complex; APCs: antigen presenting cells; Nrf-2: nuclear factor erythroid-2; WBC: white blood cell; CNS: central nervous system; i.v.: intravenous.

**Table 2:** Myelin protein/peptide-based vaccination.

Vaccine	Antigen	Targeting Ligand/Drug	Vaccination Type	Admin. Route	Admin. Dose	Animal Model	Vaccination Outcome
**Myelin Proteins/Peptides**							
MBP [112]	Guinea pig MBP	-	Prophylactic: seven days b.i.	e.c.		SJLxB10.PL female mice (6–8 weeks old) with EAE induced with MBP	Protection from RR form of EAE Reduction of disease incidence to 58%
MBP [113]	Guinea pig MBP	-	Prophylactic: seven and three days b.i. Therapeutic: at initial signs of EAE and after four days	e.c.		B10.PL female mice (6–8 weeks old) with EAE induced with MBP	Prophylactic vaccine: protection from EAE Therapeutic vaccine: suppression of EAE
MBP [114]	Guinea pig MBP	-	Prophylactic: seven and three days b.i.	e.c.		B10.PL and SJLxB10.PL female mice (6–8 weeks old) with acute or RR EAE respectively, induced with MBP Knock out mice: TCRδ^_/_^, CD1d^_^/^_^ and β_2_m^_^/^_^ on H-2^u^ background.	Vaccination with MBP prior to EAE induction prevented the development of the disease (incidence reduction by 50%) and reduced the severity of the clinical symptoms in the mice that developed EAE. Experiments with knock out mice showed that the disease could not be completely suppressed only in β_2_m^_^/^_^ mice.
MOG_35–55_ [115]	MOG_35–55_	-	Preclinical/Therapeutic: 3, 5, and 7 days p.i.	i.v.		C57BL/6 female mice (8–10 weeks old) with EAE induced with MOG_35–55_	Dramatic suppression of EAE development
c-MOG_35–55_ [116]	MOG_35–55_ and cyclic- MOG_35–55_	-	Preclinical/Therapeutic on the same day with immunization and seven days p.i.	s.c.		C57BL/6 female mice (6–10 weeks old) with EAE induced with MOG_35–55_	Amelioration of EAE clinical course and pathology. Reduction of clinical severity of acute phase of EAE and reduction of overall EAE burden.
ATX-MS-1467 [117]	Mixture of MBP_30–44_, MBP _131–145_, MBP_140–154_, MBP_83–99_	-	Prophylactic Preclinical/Therapeutic	s.c.	100 μL of ATX-MS-1467 twice a week	(ObxDR2)F1 mice with EAE induced with spinal cord homogenate	ATX-MS-1467 was shown to effectively prevent and treat EAE. The inhibition of the disease was found to be dose-dependent.
Pool of MBP peptides [118]	MBP_68–86_ and MBP_87–99_		Therapeutic: secen and 11 days p.i.	i.n.	500 μg of each MBP peptide /rat	Lewis female rats (9 weeks old) with EAE induced with MBP_68–86_	Tolerization to a pool of MBP peptides was found to result in amelioration of clinical symptoms of EAE.
MOG_35–55_ [119]	MOG_35–55_	-	Prophylactic: every other day, for 10 days b.i.	oral	200 μg of MOG_35–55_	C57BL/6 male mice (6–8 weeks old) with EAE induced with MOG_35–55_.	Oral vaccination with MOG_35–55_ was found capable of efficiently suppressing pathogenic cells.
MBP [120]	MBP	-	Prophylactic: one day b.i.	oral	100 mg of MBP	Euthymic and adult thymectomized Tg mice with EAE induced with MBP.	Euthymic Tg mice were shown to be protected from EAE after oral administration of MBP contrary to thymectomized mice, thus indicating the key role of thymus in oral tolerance induction.
**Altered peptide ligands (APLs)**							
APL [121]	P1: MBP_87–99_, P2: (Ala^91^,Ala^96^)MBP_87–99_ P3: cyclo(87–99) (Ala^91^,Ala^96^)MBP_87–99_	-	Prophylactic: on the day of immunization	s.c.		Female Lewis rats (6–8 weeks old) with EAE induced with MBP_74–85_	Suppression of EAE was detected 8 days post P2 and P3 administration. P1 was not found to suppress EAE. P2 was shown to suppress EAE between 8–16 days whereas P3 suppressed EAE until the end of the experiment (e.g., day 18 or 20).
APL [87]	[Ala^41^]MOG_35–55_, [Ala^41,46^]MOG_35–55_ and [TyrOMe^40^]MOG_35–55_ cyclo(46–55)MOG_35–55_ and cyclo(41–55)MOG_35–55_	-	Prophylactic: on the day of immunization.	s.c.		C57BL/6 female mice (12–18 weeks old) with EAE induced with rat MOG_35–55_	Significant reduction of EAE incidence and symptons with the administration of [Ala^41,46^]MOG_35–55_ or [Ala^41^]MOG_35–55_ as compared with the delivery of [TyrOMe^40^]MOG_35–55_, cyclo(46–55)MOG_35–55_ and cyclo(41–55)MOG_35–55_
**Y-MSPc**							
Y-MSPc [94]	MOG_34–56_ MBP_89–104_ OSP_55–80_ OSP_179–201_ MOBP_15–36_ PLP_139–151_ PLP_178–191_	-	Preclinical/Therapeutic: 3, 5, 7, and 21 days p.i.	i.v.	75 μg of Y-MSPc/mouse	SJL/J female mice (2–3 months old) with EAE induced with PLP_139–151_	Y-MSPc was revealed to be more efficient in inhibiting the development of the disease and suppressing its progression in comparison with a single encephalitogenic peptide or a cocktail of peptides.
Y-MSPc [93]	OSP_55–74_ MOBP_55–77_ MOBP_15–36_ MOG_34–56_ PLP_175–194_ PLP_139–151_ MBP_89–104_		Preclinical/Therapeutic: administration post immunization	i.v.	75 μg of Y-MSPc/mouse	(C57Bl/6J6SJL/J)F1 mice with EAE induced with PLP_139–151_ or rhMOG (active classical EAE), or a mixture of hMOG _34–56_, hPLP _139–151_, hMOBP_15–36_, hMBP_89–104_, hOSP_55–80_ (active complex EAE), or via transfer of line T cells specific for phMOG_34–56_ or phPLP_139–151_ (passive EAE)	Y-MSPc was shown to be more efficient in inhibiting the development of classical or complex EAE, suppressing the disease course and reversing the chronic disease, compared with a single encephalitogenic peptide or a cocktail of peptides. Additionally, Y-MSPc appeared to be more effective in suppressing passive EAE.
**Cytokine-neuroantigen (NAg) fusion proteins**							
GMCSF-NAg and MCSF-NAg [60]	Guinea pig MBP_69–87_	GM-CSF M-CSF cytokines	Therapeutic: Exp.1: 9, 10, 12, and 14 days p.i.; exp. 2: 10, 11, and 13 days p.i.; exp. 3: eight and 11 days p.i.	s.c.	1 nmol of fusion protein(s) per injection (exp. 1 and 2), 4 nmol on day 8 and 1 nmol on day 11 (exp. 3)	Lewis rats with EAE induced with DHFR-NAg fusion protein	GMCSF-NAg was found to potently target MBP_69–87_ to subsets of myeloid APCs and to successfully induce antigen-specific tolerance.
GMCSF-NAg MCSF-NAg [98]	MBP_69–87_	GMC-SF MCSF	Prophylactic: 21, 1,4 and 7 days b.i. Therapeutic: 9, 10, 12 and 14 days p.i. (exp. 1), or 10, 11, and 13 days p.i. (exp. 2), or eight and 11 days p.i. (exp. 3)	s.c.	Prophylactic: 4 nmol of fusion protein(s) per injection Therapeutic: 1 nmol (exp. 1 & 2), 4 nmol on day 8 and 1 nmol on day 11 (exp. 3)	Lewis rats with EAE induced with DHFR-NAg fusion protein	Prophylactic vaccination with GMCSF-NAg resulted in attenuation of EAE severity. Furthermore, treatment with GMCSF-NAg successfully inhibited EAE progression to more severe stages.
GMCSF-NAg [122]	MOG_35–55_	GM-CSF	Preclinical/Therapeutic: p.i.	s.c.	2 or 1 nmol of GMCSF-NAg	C57BL/6 mice with EAE induced with MOG _35–55_ (active EAE) or with activated MOG-specific Th1 T cells (passive EAE). SJL mice with EAE induced with PLP_139–151_. B cell deficient, CD4-deficient, IFN-γR1-deficient, and 2D2	GMCSF-NAg was shown to suppress the established disease especially in passive EAE models. It also proved to be an efficient therapy for *Cd4*−defficient mice and to exhibit tolerogenic activity in B cell deficient mice.
Cytokine-NAg [97]	MOG_35–55_ PLP_139–151_	GM-CSF	Prophylactic: 21, 14 and 7 days b.i. Therapeutic: 13, 15, 17, and 20 days p.i.	s.c.	Prophylactic: 2 nmol of cytokine-NAg Therapeutic: 4 nmol on days 9 and 11, and 2 nmol on day 14 p.i.	C57BL/6 with EAE induced with MOG_35–55_ (active EAE) or with transfer of activated MOG_35–55_-specific T lymphocytes. In order to provoke another bout of EAE on day 42, mice were challenged with MOG_35–55_. SJL mice with EAE induced with PLP_139–151_.	Fusion of GM-CSF with myelin protein epitopes was found to lead to efficient antigen uptake by myeloid APCs resulting in blocking of the development and progression of EAE.
Cytokine-NAg [96]	MBP_69–87_ MBP_73–87_ PLP_139–151_ MOG_35–55_	GMCSF IFN-β IL16 IL2	Prophylactic: 21, 14, and 7 days b.i. Therapeutic: 13, 15, 17, and 20 days p.i. or alternatively after the onset of paralysis	s.c.		C57BL/6 mice with EAE induced with MOG_35–55_. SJL mice with RR EAE induced with PLP_139–151_. Lewis rats with EAE (acute monophasic form) induced with MBP_73–87_	The developed cytokine-NAg fusion proteins were shown to target APCs and to successfully prevent the induction of EAE when administered prophylactically as well as to suppress on-going EAE.
Cytokine-NAg [123]	Guinea pig MBP	rat IL-2 or IL-4	Prophylactic: 21, 14 and 7 days b.i. Preclinical/Therapeutic: five days p.i. and on every other day through days 9, 11, or 13 p.i.	s.c.	Prophylactic: 0.5-1 nmol per injection	Lewis rats with EAE induced with guinea pig MBP fusion protein	Prophylactic or therapeutic vaccination with IL-2/NAg resulted in attenuation of EAE course, whereas administration of IL4-NAg indicated lack of tolerogenic activity.
GMCSF-NAg [95]	MOG_35–55_	GM-CSF	C57BL/6 mice: Prophylactic 21, 14, and 7 days b.i. 2D2-FIG mice: Preclinical/Therapeutic: 0, 7, and 14 days, or 7 and 14 days, or 14 days p.i.	C57BL/6 mice: s.c. 2D2-FIG mice: i.v.	C57BL/6 mice: 2 nmol GMCSF-MOG_35–55_ per injection 2D2-FIG mice: 4 nmol per injection	C57BL/6 mice with EAE induced with MOG _35–55_ 2D2-FIG mice with a transgenic MOG-specific repertoire of T cells and a GFP reporter of FOXP3 expression	The pretreatment with the GMCSF-MOG fusion protein elicited CD25+ Tregs which were required for the induction of tolerance. Vaccination of 2D2-FIG with GMCSF-MOG elicited circulating FOXP3+ Tregs the number of which was maintained with multiple boosters.
MOG_35–55_/I-A^b^ dimer [107]	MOG_35–55_	I-A^b^ dimer	Therapeutic: nine days p.i. (treatment duration: four days).	i.p.	12 nM MOG_35–55_/I-A^b^ dimer (1 μg/mouse/day)	C57BL/6 female mice (6–8 weeks old) with EAE induced with MOG_35–55_	The administration of MOG_35–55_/I-A^b^ dimer resulted in the reduction of antigen-specific T cells and amelioration of EAE symptoms.
**Antibodies coupled with myelin peptides**							
α-receptor–MOGp mAbs [100]	DNA for MOG_29–59_ (MOGp)	α-DEC mAbs α-Langerin mAb	Prophylactic: transfer of MOG-specific CD4+ T cells 15 days b.i. and admin. of α-receptor–MOGp mAbs 14 days b.i.	s.c.	3 μg of α-receptor mAbs	C57BL/6 (B6) mice with EAE induced with MOG_35–55_	Prophylactic vaccination with α-DEC- and a-Langerin–MOGp mAbs led to reduction of disease incidence, onset delay and amelioration of clinical scores.
αDEC205-PLP_139–151_ mAb [Stern et al., 2010]	PLP_139–151_	anti-DEC205	Prophylactic: 10 or 15 days b.i.	i.p.	1 μg of fusion mAb	SJL/J female mice (6–10 weeks old) with EAE induced with PLP _139–151_	Administration of αDEC205-PLP_139–151_ mAb was found to alleviate the disease symptoms.
scFv DEC:MOG [102]	MOG	scFv specific for DEC205	Prophylactic: seven and three days b.i. Therapeutic: oje and four days after disease onset, signified by a clinical score equal to 1	i.v.	10 μg of scFvDEC:MOG	C57/Bl6 mice with EAE induced with WSCH	Almost complete prevention of EAE (90% of mice) was observed by administration of scFv DEC:MOG b.i. Moreover, vaccination with scFv DEC:MOG p.i. resulted in significant alleviation of the clinical symptoms in 90% of the mice.
αDCIR2-PLP_139–151_ fusion mAb [79]	PLP_139–151_	αDCIR2	Prophylactic: 10 days b.i.	i.p.	1 μg of fusion mAbs	SJL/J female mice (6–10 weeks old) with EAE induced with PLP_139–151_ (active EAE) or via adoptive transfer of splenocytes from αDCIR2-PLP_139–151_-treated mice (passive EAE)	Vaccination with αDCIR2+-PLP_139–151_ fusion mAb was shown to decrease the severity of the disease and to delay its onset. Mice receiving splenocytes from αDCIR2-PLP_139–151_-treated mice exhibited substantially lower clinical scores in comparison to those receiving cells from αDCIR2 mAb-treated mice.
αCD4/CD8+PLP_139–151_ [103]	PLP_139–151_	Anti-CD4, anti-CD8a Ab	Prophylactic: admin. of mAb 21 days b.i. followed by PLP_139–151_ delivery every other day for 16 days. Therapeutic: Mice treated with αCD4/CD8 Abs on day 11 p.i. were injected with αCD4/CD8+PLP_139–151_ every other day from day 12–26.	i.p.	100 μg of CD4-/mouse) 100 μg of CD8a-/mouse 25 μg PLP_139–151_ per injection	SJL female mice (seven weeks old) with EAE induced with PLP_139–151_	αCD4/CD8+PLP_139–151_-treated mice exhibited substantially lower EAE scores and reduced rate of relapses in chronic disease
**Recombinant T-cell receptor ligands (RTLs)**							
RTL342M [124]	MOG_35–55_	HLA-DR2 peptide-binding domains	Therapeutic (s.c. or i.v.): admin. on the day that the clinical score for each mouse was ≥ 2. Daily admin. for mice receiving multiple doses. Prophylactic (s.c.): admin. of 4, 9, or 14 doses within 15 days. EAE was induced 2 days after the admin. of the final dose.	i.v. s.c.	50 μg of RTL342M	HLA-DR2 positive male/female mice (8–12 weeks old) with EAE induced with MOG_35–55_	RTL treatment was revealed to be more efficient in reducing paralysis when administered in the form of multiple doses instead of a single dose, independently of the administration mode. Furthermore, the treatment with RTL342M could treat or prevent relapses. Pretreatment with RTL342M was shown to prevent the disease.
RTL401 [125]	PLP_139–151_	*α*1 and *β*1 domains of the I-A^s^ class II molecule	Upon EAE onset, daily i) i.v. admin. for 3–4 days and ii) s.c. admin. for 8 days.	i.v. s.c.	100 μg of RTL401	SJL mice (6–7 weeks of age) with EAE induced with PLP_139–151_ or PLP_178–191_ or MBP_84–104_. C57BL/6 X SJL) F1 mice (6–7 weeks of age) with EAE induced with MOG_35–55_ or PLP_139–151_.	i.v. or s.c. vaccination with RTL401 resulted in prevention of relapses and long-term reduction of clinical severity only in SJL mice and C57BL/6 X SJL) F1 mice with EAE induced with PLP_139–151_.
RTL401 [126]	PLP_139–151_	*α*1 and *β*1 domains of the I-A^s^ class II molecule	Upon EAE onset, daily (i) i.v. admin. for five days and (ii) s.c. for eight days.	i.v. s.c.	100 μL of 1 mg/mL RTL401	SJL female mice (7–8 weeks old) with EAE induced with PLP_139–151_ (active EAE) or via transfer of activated PLP_139–150_-specific T cells (passice EAE)	i.v. or s.c. vaccination with RTL401 was shown to effectively discontinue passive EAE progression, reverse its clinical severity and reduce the infiltration of cells into the CNS, as in the treatment of active EAE. Injury to axons was also prevented.
RTL551 [127]	MOG_35–55_	*α*1 and *β*1 domains of the I-A^b^ class II molecule	Upon EAE onset (days 12–14 for active EAE and days 7–12 for passive EAE), daily i.v. admin. for five days.	i.v.	100 μL of 1 mg/mL RTL551	C57BL/6 male mice (6–7 weeks of age) with EAE induced with MOG_35–55_ (active EAE) or via transfer of activated cells (passive EAE).	RTL551 treatment of actively or passively induced EAE resulted in significant reduction of clinical symptoms and spinal cord lesions.
RTL401, RTL402, RTL403 [128]	PLP_139–151_ PLP_178–191_ MBP_84–104_	*α*1 and *β*1 domains of the I-A^s^ class II molecule	At EAE onset (days 10-11), when the clinical score was ≥2, daily s.c. admin. for 8 days.	s.c.	100 μL of 1 mg/mL RTL	SJL/J female mice (7–8 weeks old) with EAE induced with WSCH or with a mixture of PLP_139–151_ and PLP_178–191_.	A single RTL was found capable of successfully treating ongoing disease induced with a mixture of encephalitogenic epitopes as long as the cognate T cell specificity was present.
RTL551 [106]	rhMOG, hMOG_35–55_, mMOG_35–55_	*α*1 and *β*1 domains of the I-A^b^ class II molecule	At EAE onset (days 10–13), when the clinical score was ≥2, daily i.v. admin. for eight days.	i.v.	100 μL of 1 mg/mL RTL551	C57BL/6 male mice (7–8 weeks old) with EAE induced with rhMOG or mMOG_35–55_.	Vaccination with RTL551 could reverse the progression of EAE, reduce demyelination and damage of axons without however induce suppression of anti-MOG Ab response.
RTL401 [129]	PLP_139–151_	*α*1 and *β*1 domains of the I-A^s^ class II molecule	Upon EAE onset (days 10–11), daily admin. for 1, 2, or 5 days.	s.c.	100 μL of 1 mg/mL RTL401	SJL/J female mice (7–8 weeks old) with EAE induced with PLP_139–151_ (active EAE) or via transfer of activated cells (passive EAE). TCR Tg 5B6 mice with EAE induced with PLP_139–151_ B cell deficient (μMT knock-out, KO) mice on C57BL/6 background (7–8 weeks old) with EAE induced with MOG_35–55_.	A new interaction between cells was revealed via which the RTL-equipped myeloid APCs reverse EAE progression by transferring tolerogenic signals to cognate T lymphocytes. It was also found that splenocytes incubated with RTL401 exhibited reduced ability to passively transfer EAE. Finally, it was shown that EAE can be treated by RTL551 in the absence of B cells.
VG312, VG303, VG311 [108]	MOG_35–55_, MBP_85-99_, CABL	*α*1 and *β*1 domains of DR2	Therapeutic: i.v. administration for eight consecutive days, 2–4 days after the disease onset.	i.v.	100 μL of VG312, VG303, VG311	Tg HLA-DR2 male and female mice (8–12 weeks old) with EAE induced with MOG_35–55_	Vaccination with VG312 led to peptide- and dose-dependent induction of long-term tolerance to the encephalitogenic epitope MOG_35–55_ and reversal of the clinical/histological symptoms of EAE
RTL401 [130]	PLP_139–151_	*α*1 and *β*1 domains of the I-A^s^ class II molecule	Therapeutic: (i) i.v. admin. for five consecutive days (days 20–24) and (ii) s.c. admin. for 3 days (days 32–34).	i.v. s.c.	100 μg of RTL401	SJL/J female mice (7–8 weeks old) with EAE induced with PLP_139–151_.	Administration of RTL401 post the relapsing EAE peak resulted in prevention of disease relapses, reduction of demyelination and axonal damage.
**Bifunctional peptide inhibitor (BPI)**							
PLP-B7AP [131]	PLP_139–151_	B7 antisense peptide (AP) derived from CD28 receptor	Prophylactic 11, 8, and 5 days b.i. Preclinical/Therapeutic: 4, 7, and 10 days p.i.	s.c.	Prophylactic: 50 or 100 nmol PLP-B7AP/injection Therapeutic: 100 nmol PBI/injection	SJL/J female mice (5–7 weeks old) with EAE induced with PLP_139–151_	Both prophylactic and therapeutic vaccination with PLP-B7AP resulted in efficient suppression of EAE. Mice treated with PLP-B7AP exhibited significantly low demyelination.
PLP-LABL [132]	PLP_139–151_	LABL	Prophylactic: 11, 8, and 5 days b.i.	s.c.	100 nmol/injection/day	SJL/J female mice (5–7 weeks old) with EAE induced with PLP	The vaccination with PLP-LABL inhibited the inflammatory response resulting in prevention of BBB disruption and thus inhibition of EAE onset and progression.
PLP-LABL derivatives [110]	PLP_139–151_	LABL	Therapeutic: admin. on disease onset, signified by a clinical score ≥1, and for three consecutive days until the score was <1)	i.v.	100 nmol/mouse	SJL/J (H-2S) female mice (5–7 weeks old)	Vaccination with the synthesized BPI derivatives was shown to efficiently inhibit EAE severity, and incidence.
PLP-LABL [133]	PLP_139–151_	LABL	Preclinical/Therapeutic: 4, 7, 10, and 14 days p.i.	i.v.	100 mol/mouse	SJL/J female mice (5–7 weeks old) with EAE induced with PLP_139–151_	Low disease scores and incidence could be observed in mice vaccinated with PLP-LABL.
PLP-LABL derivatives [134]	PLP_139–151_	LABL	Therapeutic: admin. on disease onset, signified by a clinical score ≥1, and for three consecutive days until the score was <1)	i.v.	100 nmol/mouse	SJL/J female mice (5–7 weeks old) with EAE induced with PLP_139–151_	The synthesized BPI derivatives were revealed to suppress EAE progression after intravenous administration more efficiently in comparison with unmodified BPI.
BPI-Fc fusion peptides LABL-Fc-ST-PLP and LABL-Fc-ST-MOG [109]	PLP_139–151_ MOG_38–50_	LABL-Fc-ST	Preclinical/Therapeutic: four and seven days p.i.	i.v.	25 nmol per dose	SJL/J mice (5–7 weeks old) with EAE induced with PLP_139–151_	BPI-Fc fusion peptides were revealed to be highly efficient in suppressing EAE. The vaccinated mice were not found to exhibit weight loss, and featured benign clinical symptoms and reduced demyelination.
PLP–cIBR Derivatives [135]	PLP_139–151_	cIBR7 peptide	Studies I and II: 4, 7, and 10 days p.i. Study III: admin. on disease onset, signified by a clin. score ≥1, and for 3 consecutive days until the score was <1	i.v.	Study I: 100 nmol/injection/day Study II and III: 50 nmol/injection/day	SJL/J (H-2S) female mice (5–7 weeks old) with EAE induced with PLP_139–151_	Vaccination with PLP–cIBR, even at low dose or less frequent i.v. injections, resulted in significant amelioration of EAE and protected CNS against demyelination.
Multivalent BPI (MVB_MOG/PLP_) [111]	MOG_38–50_ PLP_139–151_	LABL	Preclinical/Therapeutic 4, 7, and 10 days p.i.	s.c.	100 nmol/mouse	SJL/J female mice (5–7 weeks old) with EAE induced with PLP_139–151_ C57BL/6 mice (4–6 weeks old) with EAE induced with MOG_38–50_	MVB_MOG/PLP_ was found to significantly suppress EAE in both animal models despite the evidence of epitope spreading in the C57BL/6 mice.
**Antigen-drug conjugates**							
PLP_139−151_-DEX [61]	PLP_139−151_	DEX	Preclinical/Therapeutic: 4, 7, and 10 days p.i.	s.c.		SJL/J female mice (4–6 weeks old) with EAE induced with PLP_139–151_	Vaccination with PLP_139–151_-DEX efficiently protected the SJL/J mice from the onset of clinical symptoms compared with DEX treatment.

MBP: myelin basic protein; b.i.: before immunization; EAE: experimental autoimmune encephalomyelitis; e.c.: epicutaneous; RR: relapsing-remitting; MOG: myelin oligodendrocyte glycoprotein; p.i.: post immunization; i.n.: intranasal; APL: altered peptide ligand; Y-MSPc: recombinant synthetic protein comprising multiple epitopes of the human myelin protein; OSP: oligodendrocyte-specific protein; MOBP: myelin associated oligodendrocyte basic protein; PLP: proteolipid protein; GMCSF: Granulocyte-macrophage colony-stimulating factor; MCSF: macrophage colony stimulating factor; DHFR: dihydrofolate reductase; i.p.: intraperitoneal; IFN: interferon; IL: interleukin; mAbs: monoclonal antibodies; scFv: single chain fragment variables; WSCH: whole spinal cord homogenate; RTL: recombinant T-cell receptor ligand; HLA: human leucocyte antigen; rhMOG: recombinant human MOG; mMOG: murine MOG; BPI: bifunctional peptide inhibitor; LABL: ICAm-I binding peptide; DEX: dexamethasone.

**Table 3:** DNA vaccination.

Vaccine	Antigen/Immunosuppr.	Vaccination Type	Admin. Route	Admin. Dose	Animal Model	Vaccination Outcome
pDNA encoding IL-4 pDNA encoding PLP_139–151_ pDNA encoding *MOG* [142]	PLP_139–151_	Prophylactic: 17 and 10 days b.i. Therapeutic: 14 and 21 days p.i Co-vaccination with IL-4 plasmid and MOG plasmid on days 18 and 27 p.i.	i.m	100 μg of plasmid per injection	SJL/J mice with EAE induced with PLP_139–151_ C57BL/6 mice with EAE induced with MOG_35–55_	Co-vaccination with IL-4 and PLP_139–151_ plasmids significantly protected against induction of EAE. Co-vaccination with IL-4 plasmid and MOG plasmid reversed ongoing EAE.
pMOG _91–108_ pK0-MOG_91–108_ (lacking CpG motifs) [143]	MOG_91–108_	Prophylactic: three weeks b.i.	i.m.	200 μg DNA/injection	LEW.1AV1 (RT1av1) female rats (4–5 weeks old) with EAE induced with MOG_91–108_	Vaccinated rats were protected against EAE.
pDNA encoding IL-10 pDNA encoding MBP_68–86_ [144]	MBP_68–86_	Admin. at the disease onset			Female Lewis rats (~6 weeks old) with EAE induced with MBP_68–86_ or MBP_87–99,_ or with EAN induced with P2_57–81_	Rats co-vaccinated with IL-10 and MBP_68–86_ plasmids went into rapid remission. Co-administration of pDNA encoding IL-10 and pDNA encoding MBP_68–86_ were shown to suppress EAE in rats induced either with MBP_68–86_ or MBP_87–99_ but not EAN.
pZZ/MOG_91–108_ pMOG_91–108_ pK0-MOG_91–108_ pK3-MOG_91–108_ [145]	MOG_91–108_	Prophylactic: 3–4 weeks b.i.	i.m.	200 μg DNA/injection 100 μg of CpG DNA were added to pMOG_91–108_ before the injection	Female LEW.1AV1 (RT1av1) rats (4–5 weeks old) and female DA rats with EAE induced with MOG_91–108_	Vaccination with pDNA encoding MOG_91–108_ (lacking the ZZ gene) reduced clinical symptoms of EAE and mortality in rats with different genetic background sharing the same MHC.
DNA encoding MBP, PLP, MOG, MAG and IL-4- [10]	MBP, PLP, MOG, MAG/GpG ODN	Therapeutic: admin. at the peak of acute EAE, when mice exhibited paralysis	i.m. i.p.	0.025 mg of each myelin peptide plasmid, 0.05 mg of IL-4 plasmid and 0.05 mg of GpG ODN	Female SJL/J and C57BL/6 (B6) mice (8–12 weeks old) with EAE induced with PLP_139–151_ or MOG_35–55_	Administration of myelin cocktail/IL-4 plasmids and the immunosuppressant GpG ODN resulted in dramatic improvement of the disease in mice having either chronic relapsing or chronic progressive EAE.
pMOG _91–108_ pMOG-IFN-β pMOG-scr [146]	MOG_91–108_	Prophylactic: three weeks b.i.	i.m.	200 μg DNA/injection	Female LEW.1AV1 (RT1av1) rats (4–5 weeks old) and female DA rats with EAE induced with MOG_91–108_	The suppressive ability of DNA vaccination was found to be abrogated via silencing IFN-β.
p2MOG35 [147]	MOG_35–55_/Tacrolimus (FK506)	Preclinical/Therapeutic: three and 17 days p.i.	i.m.	100 μg of p2MOG35/mouse 10 μg of FK506/mouse	Female C57BL/6 mice (6–8 weeks old) with EAE induced with MOG_35–55_	Co-administration of p2MOG35 with FK506 was shown to effectively meliorate EAE in mice.
pVAX-PLP, pVAX-MOG [148]	PLP, MOG	Prophylactic: four or 12 weeks b.i.	i.m.	20μg pVAX-PLP, pVAX-MOG	Female SJL/J (9H-2) mice (6 weeks old) with EAE induced with PLP_139–151_ C57/B6 mice with EAE induced with MOG_35–55_	EAE was found to be exacerbated in mice vaccinated with pVAX-PLP 4 weeks prior to immunization whereas both clinical and pathological symptoms were suppressed in mice vaccinated 12 weeks prior to EAE induction. In mice vaccinated with pVAX-MOG, either four or 12 weeks prior to immunization, EAE was shown to be significantly suppressed.

pDNA: plasmid DNA; IL: interleukin; MOG: myelin oligodendrocyte glycoprotein; b.i.: before immunization; p.i.: post immunization; PLP: proteolipid protein; i.m.: intramuscular; EAE: experimental autoimmune encephalomyelitis; MBP: myelin basic protein; EAN: experimental autoimmune neuritis; i.p.: intraperitoneal; GpG: GpG oligonucleotide; DA rats: dark agouti rats; IFN: interferon; pVAX: expressing vector.

**Table 4:** Cell-based vaccination.

Cells	Inductive Agent/Peptide	Vaccination Type	Admin. Route	Admin. Dose	Animal Model	Vaccination Outcome
**Tolerogenic Dendritic cells (tolDCs)**						
BMDCs from C57BL/6 mice [161]	Atorvastatin/MOG_35–55_	Preclinical/Therapeutic: days five and 13 p.i.	i.p.	1 × 10^6^ cells per injection	Female C57BL/6 mice (8–10 weeks old) with EAE induced with MOG_35–55_	MOG_35–55_—specific tolDCs successfully ameliorated clinical Symptoms in mice with EAE.
BMDCs [162]	mytomycin C/MOG_196–204_	Admin. of MOG196-pulsed Kb−/−Db−/− DCs to C57BL/6 (B6) mice one week b.i. and one p.i. Admin. of MOG196-pulsed B6 DCs to C57BL/6 mice three days b.i. and two and seven days p.i.	s.c.	1 × 10^6^ cells per injection	Female C57BL/6 (B6) (8–10 weeks old) with EAE induced with MOG_35–55_	Administration of MOG196-pulsed Kb−/−Db−/− DCs or MOG196-pulsed DCs ameliorated EAE in mice.
Murine BMDCs [154]	1α, 25-dihydroxy-vitamin D3/MOG-encoding mRNA or MOG_35–55_	Therapeutic: 13, 17, and 21 days p.i.	i.v.	1 × 10^6^ cells per injection	Female C57BL/6JOlaHsd mice (8–10 weeks old) with EAE induced with MOG_35–55_	Vaccination with tolDCs electroporated with MOG-encoding mRNA or MOG_35–55_ stabilized the clinical signs of the disease already from the first injection. MRI examination of hyperintense spots present along the spinal cord of mice was found to be in line with the clinical score (Figure 9).
BMDCs [163]	CD40-specific and p19-specific shRNA encoding lentiviral vectors/pyromycin/MOG_35–55_	Preclinical/Thereapeutic: 3, 5, and 7 days p.i.	i.v.	2 × 10^6^ cells per injection	C57BL/6 mice with EAE induced with MOG_35–55_	Administration of MOG35–55-pulsed and lentiviral transduced BMDCs led to significant decrease in the clinical symptoms of EAE in mice. The highest decrease in the clinical scores was observed with the administration of co-transduced BMDCs (BoLV-DCs).
BMDCs [164]	Vitamin D3/MOG_40–55_	Preclinical/Therapeutic: two and five days p.i., or five and nine days p.i. or 15, 19, 23, and 33 days p.i.	i.v.	2 or 4 × 10^6^ cells	Female C57BL/6J mice (8–10 weeks old) with EAE induced with MOG_40–55_	MOG_40–55_—specific TolDCs were found to succeed in reducing EAE incidence and ameliorating its clinical signs.
BMDCs [165]	Vitamin D3/MOG_40–55_/cryopreserved		i.v.	2 or 4 × 10^6^ cells	Female C57BL/6J mice (8–10 weeks old) with EAE induced with MOG_40–55_	It was shown that MOG_40–55_—specific TolDCs maintain their tolerogenic properties and can efficiently ameliorate the clinical symptoms of EAE.
Murine BMDCs [166]	Tofacitinib/MOG_35–55_	Therapeutic: 7, 11, and 15 days p.i.	i.v.		Twelve-week Female C57BL/6 mice (12 weeks old) with EAE induced with MOG_35–55_	MOG_35–55_—specific TolDCs efficiently dampened EAE severity and progression.
BMDCs [167]	1,25-dihydroxyvitamin D_3_/MOG_35–55_	Therapeutic: 10, 13, and 16 days p.i.	i.v.		Female C57BL/6 mice (6–8 weeks old) with EAE induced with MOG_35–55_	Vitamin D3 treated MOG_35–55_—specific. TolDCs succeeded in postponing the disease onset and reducing its clinical scores.
DCs [168]	Estriol (E3)/MOG_35–55_	Prophylactic: one day b.i.	i.v.	8–10 × 10^6^ cells per mouse	Female C57BL/6 (H-2b) mice (4–6 weeks old) with EAE induced with MOG_35–55_	Mice vaccinated with E3 MOG_35–55_—specific TolDCs exhibited a reduced cumulative clinical score and EAE severity. They also avoided relapses and development of chronic disease.
BMDCs matured with TNF-α [169]	/MOG_35–55_	Prophylactic: 7, 5, 3, and 1 days b.i. Preclinical: one day p.i.	i.v.	2–2.5 × 10^6^ cells per injection Rat anti–mouse IL-10R mAb: 0.5 mg equivalents per mouse	C57Bl/6 mice with EAE induced with MOG_35–55_	Vaccination with MOG_35–55_—specific TNF/DCs improved the clinical disease score. Pulsing of TNF-α/DCs with an unrelated peptide did not succeed in preventing the disease.
DCs [170]	/in vivo pulsing in Lewis rats with EAE induced with MBP_68–86_	Prophylactic: four weeks b.i.	s.c.	1 × 10^6^ cells per rat	Male Lewis rats with EAE induced with MBP_68–86_	Injection of EAE DCs to rats resulted in induction of immune tolerance against the disease as demonstrated by delayed onset and marked decrease of the mean clinical score.
**T cell-based vaccination**						
Ob2F3 Tregs [171]	Retrovirally transduced pre-stimulated naïve CD4+ Tcells from peripheral blood mononuclear cells (PBMCs) of healthy donors using Ob2F3.	Preclinical/Therapeutic: seven days p.i.	i.v.	2 × 10^6^ cells	Male and female HLA-DR15 transgenic mice (4.5–7.5 months old) with EAE induced with MOG_35–55_	Ob2F3 Tregs were shown to significantly ameliorate MOG_35–55_ induced EAE via bystander suppression.
MBP-specific T-cell lines (e.g., B12 and B12-GFP) [157]		Prophylactic: admin. three times at weekly intervals, with the last injection 10 or seven days b.i.	s.c.	1 × 10^7^ activated and irradiated T cells	Female Lewis rats (6–8 weeks old) with EAE induced via i.v. injection of antigen stimulated T cells.	Vaccination with MBP-specific T cell lines inhibited the development of EAE clinical symptoms.
**Hematopoietic stem cells (HSCs)**						
DC-MOG vector-transduced BM-HSC [172]	Ex vivo modification of HSCs with SIN lentivirus vectors which transcriptionally target the expression of myelin peptides to DCs.	Prophylactic: Lethally Irradiated (10.5 Gy) mice were transplanted with DC-MOG transduced BM-HSCs eight weeks b.i. BM chimeras received neomycin treatment for three weeks post transplantation.	i.v.	1–3 × 10^6^ cells per mouse	C57BL/6 mice with EAE induced with MOG peptide.	The transplantation of DC-MOG vector-transduced BM-HSC was found to completely protect mice from developing EAE even in cases of transplantation 6 months b.i. In agreement with the clinical observations, no histological signs of the disease such as demyelination, damage of axons, etc. could be detected in the tolerized mice.
**Bone marrow cells (BMC)**						
BMCs expressing MOG_40–55_ [173]	liMOG	Prophylactic: mice were transplanted with BMCs transduced with liMOG 21 days b.i. Therapeutic: mice were transplanted with transduced BMCs 15–17 days p.i.	i.v.	0.7–1.6 × 10^6^ cells per mouse	Female C57BL/6J mice (5–10 weeks old) with EAE induced with MOG_40–55_	Transplantation of BMCs expressing MOG_40–55_ was shown to protect mice from developing EAE and reduce the disease severity in mice with established EAE.
**Myeloid-derived suppressor cells (MDSCs)**						
MDSCs isolated via positive selection from BMCs expressing MOG_40–55_ [174]	liMOG	Prophylactic: mice were transplanted with MDSCs transduced with liMOG seven days b.i. Therapeutic: mice were transplanted with transduced MDSCs 13–14 days p.i.	i.v.	0.5–1 × 10^6^ cells per mouse	Female C57BL6/J mice (6–8 weeks old) with EAE induced with MOG_40–55_	MOG_40–55_ -expressing MDSCs were found to exhibit both preventive and therapeutic effects in EAE induced with MOG_40–55_
**Antigen-cell conjugates**						
Ag-SP [158]	Chemically treated Ag-coupled SPs	Administration on day −7 b.i. or at peak of disease in actively induced EAE, or two days p.i.	i.v.	50 × 10^6^ Ag-SPs per mouse	Wild-type C57BL/6 (I-Ab), B10.S (I-As), and BALB/c (I-Ad) female mice (5–6 weeks old) with EAE induced with myelin peptide or via adoptive transfer.	It was revealed that syngeneic or allogeneic Ag-SPs can effectively protect mice against ongoing clinical EAE.
Ag-SP [159]	Chemically treated Ag-coupled SPs	Prophylactic: at indicated time points b.i.	i.v.	50 × 10^6^ Ag-SPs or 15–20 μg Ag per mouse	SJL and C57BL/6 mice with EAE induced with myelin peptide or via adoptive transfer.	i.v. infusion of peptide antigens coupled to syngeneic splenic leukocytes (Ag-SP) was found to efficiently induce antigen-specific T cell tolerance.
Ag-RBC [160]	Genetically engineerd Kell-LPETGG RBCs, coupled with MOG _35–55_ through enzymatic surface modification with sortase transpeptidase.	Prophylactic: transfusion seven days b.i. Preclinical: transfusion five days p.i. Therapeutic: Transfusion on the day of EAE onset	i.v.	200 μL RBC-MOG_35–55_	C57BL/6J (CD45.2+), B6.SJL-Ptprc (CD45.1+), BALB/c Female C57BL/6 mice (10–12 weeks old) with EAE induced with MOG_35–55_	The transfusion of RBC-MOG_35–55_ was shown to significantly improve the clinical signs of EAE in mice.

BMDCs: Bone marrow-derived dendritic cells; p.i.: post immunization; i.p.: intraperitoneal; EAE: experimental allergicencephalomyelitis; tolDCs: tolerogenic dendritic cells; s.c.: subcutaneous; b.i.: before immunization; i.v.: intravenous; MBP: myelin basic protein; Tregs: regulatory T cells; Ob2F3: recombinant T-cell receptor (TCR) isolated from a MBP specific Tcell clone of a multiple sclerosis patient; HSCs: hematopoietic stem cells; SIN: selfinactivating; SP: splenocytes; RBCs: red blood cells; liMOG: vector encoding the murine invariant chain (Ii) containing MOG_40–55_ and enhanced green fluorescent protein (EGFP).

**Table 5:** Carrier-aided vaccination.

Carrier	Particle Size (nm)	Zeta Potential (mV)	Antigen	Ag Loading (wt%)/Enc. Eff. (%)	Immunomodul. Agent	Vaccination Type	Admin. Route	Dose	Animal Model	Vaccination Outcome
**Polymer particles**										
PLGA NPs [193]	-	-	MOG_35–55_	-	(r) IL-10	Prophylactic: 31 and 15 days b.i. Therapeutic: eight and 22 days p.i.	s.c.		Female C57BL/6 mice with EAE induced with MOG_35–55_	Vaccination with mixed PLGA- MOG_35–55_ and PLGA-IL10 both in a prophylactic and therapeutic setting resulted in significant protection, decrease of EAE severity and reduction of histopathological lesions in spinal cord.
PLGA NPs [194]	-	-	PLP_139–151_	8μg/mg NP	TGF-β (166ng/mg NP)	Prophylactic: seven days b.i. Therapeutic: 13 days p.i.	i.v. s.c.	2.5, 1.25, 0.0625 mg NPs	Female SJL/J mice (6–8 weeks old) with EAE induced with PLP_139–151_	i.v. vaccination with PLGA- PLP_139–151_-TGF-β demonstrated improved efficiency at lower doses. s.c. delivery of TGF-β-coupled to PLGA- PLP_139–151_ NPs reduced the severity of relapses in EAE.
PLGA MPs [195]	800, 55,000		MOG_35–55_	-/48.6	Vitamin D3 TGF-β1 Recombinant mouse GM-CSF	Preclinical/Therapeutic: 4, 7, and 10, days p.i.	s.c.		Female C57BL/6 mice (10–11 weeks old) with EAE induced with MOG_35–55_	Delivery of various immunomodulators combined with MOG_35–55_ via a dual size MP platform resulted in the induction of enhanced antigen-specific autoimmune protection.
PLGA NPs [196]	151.2, 521.7	−14.1, −5.65	MOG_35–55_	2.58, 0.96 /25.85, 9.65	-	Prophylactic: seven days b.i.	i.v. s.c.	2 mg NPs containing 20 μg MOG_35–55_	Female C57BL/6 mice (6–8 weeks old) with EAE induced with MOG_35–55_	The intravenous injection of PLGA- MOG_35–55_ was shown to delay EAE incidence and enhance antigen-specific immune tolerance.
PLGA-PEMA NPs [197]	429.9	−67.4	PLP_139–151_PLP_178–191_	0.85/10.61	-	Prophylactic: 7, 25, and 50 days b.i. Preclinical/Therapeutic: 4, 14, and 18 days p.i.	i.v. i.p. s.c. oral	0.0625 0.125 0.625 1.25	Female SJL/J mice (6-8 weeks old) with EAE induced with PLP_178–191_	Vaccination with PLP epitope-coupled PLGA-PEMA NPs was shown to both prevent and treat relapsing-remitting EAE. Tolerance induction was antigen-specific. The i.v. administration route was the most effective.
PLGA/PLA-PEG NPs [198]	-	-	PLP_139–151_		rapamycin	Prophylactic: 14 and 21 days b.i. Therapeutic: 13 days p.i.	s.c. i.v.		SJL mice with EAE induced with PLP_139–151_	s.c. vaccination with the tolerogenic NPs inhibited paralysis. Therapeutic s.c. treatment completely inhibited EAE relapses. A single therapeutic dose of tolerogenic NP sadministered i.v. near the peak of EAE resulted in complete prevention of relapses.
PLGA-PEMA NPs [199]	377.9, 621.5–834.8	−72.8, −50 to −43.7	PLP_139–151_ PLP_178–191_	0.58, 0.24–0.83/7.2, 4.4–16.5	-	Prophylactic: seven days b.i. Therapeutic: 18 days p.i.	i.v.		SJL/J mice with EAE induced with PLP_139–151_ or PLP_178–191_	Antigen-specific immune tolerance was successfully induced by PLP encephalitogenic epitopes, encapsulated in or conjugated with PLGA-PEMA NPs.
PLGA NPs [181]	217	-	MOG40–54/H-2Db-Ig dimer, MOG35–55/I-Ab multimer	-	anti-Fas, PD-L1-Fc TGF-β1CD47-Fc	Therapeutic: 8, 18, 28, and 38 days p.i.	i.v.	1 mg NPs/mouse/injection	Female C57BL/6J mice (8–10 weeks old) with EAE induced with MOG35–55	Four i.v. injections of the developed NPs resulted in long-lasting amelioration of the disease by markedly reducing neuroinflammation, clinical EAE score and demyelination
PLGA NPs PLA NPs [200]	PLGA: 351.3–436.2 PLA: 443.2	PLGA: −40.6 to −39.8 PLA: −40.2	PLP_139–151_	PLGA: 0.25–0.28 PLA: 0.25	-	Preclinical/Therapeutic: seven days p.i.	i.v.	2.5, 2.0, 1.5 or 1.0 mg NPs/mouse	Female SJL/J mice (8–10 weeks old) with EAE induced with PLP_139–151_	Low dose vaccination with PLA NPs resulted in long-lasting (>200 days post immunization) significant reduction of the clinical score at the chronic stage of EAE contrary to vaccination with PLGA NPs.
PLGA MPs PEI-coated PLGA-MPs [201]	PLGA: 5080	PLGA: 45.3	MOG_35–55_ MOG_40–54_ MOG_40–54_/H-2Db-Ig dimer, MOG35–55/I-Ab multimer		anti-Fas, PD-L1-Fc TGF-β1 CD47-Fc	Therapeutic: 8, 18, 28, and 38 days p.i.	i.v. i.p. s.c.		Female C57BL/6J mice with EAE induced with MOG_35–55_	Four injections of the multipotent particles resulted in long-lasting suppression of EAE and reduction of neuroinflammation in an antigen-specific manner.
PLGA MPs [202]	8000		Ac-PLP-BPI-NH2-2	1.4/8.2		Preclinical/Therapeutic: 4, 7, 10, and 14 days p.i.	s.c.		Female SJL/J mice (5–7 weeks old) with EAE induced with PLP139–151	Administration of PLGA MPs resulted in slightly less efficient reduction of EAE symptoms compared with the administration of the peptide solution, but without toxicity.
PLGA [203]	400–656	−51.3 to −38.0	PLP _139–151_ PLP _178–191_	0.26–0.8		Prophylactic: seven and one days b.i.	i.v.		Female SJL/J mice (6–8 weeks old) with EAE induced with PLP_139–151_ or both PLP_139–151_ and PLP_178–191_	PLGA NPs coupled with a PLP encephalitogenic epitope were shown to efficiently induce antigen-specific tolerance in a mouse model of relapsing-remitting EAE induced either by PLP_139–151_ or by both PLP_139–151_ and PLP_178–191_
PLGA NPs [204]	363–420	-	PLP_139__–151_		LABL	Preclinical/Therapeutic: 4, 7, and 10 days p.i.	s.c.	100 nmol PLP per injection	SJL/J female mice (5–7 weeks old) with EAE induced with PLP_139__–151_	It was shown that efficient suppression of EAE required the co-administration of PLP peptide and LABL.
PLGA [205]	538	−43	PLP_139–151_	0.41–0.98	-	Preclinical/Therapeutic: seven days p.i.	i.v.	1 to 100 μg/mL NPs per injection	SJL/J mice with EAE induced with PLP_139–151_	Antigen-specific, dose-dependent tolerance was successfully induced in an EAE model via the administration of PLGA NPs couple with a PLP peptide.
PLGA [206]	500	-	PLP_139–151_	-	IL2	Prophylactic: secen days b.i. Therapeutic: 11 days p.i.	i.v.		SJL/J mice with EAE induced with PLP_139–151_	Vaccination with PLGA NPs loaded with PLP_139–151_ was found to prevent EAE onset and modulate its course.
PLGA MPs [207]	3900	-	MOG_35–55_	0.73/38	Rapamycin (loading: 0.17%/enc. eff. 42.1%)	Therapeutic: 10 days p.i.	direct intra-lymph node (LN) injection	2 mg MPs per mouse or 1 mg MPs per LN	Female C57BL/6J mice (10–11 weeks old) with EAE induced with MOG_35–55_	A single intra-LN injection (at the peak of EAE) of PLGA NPs containing a MOG peptide and rapamycin was revealed to permanently reverse paralysis.
Colloidal gel based on self-assembly of PLGA-CS and PLGA-Alginate NPs [208]	PLGA-CS: 400.1, PLGA-Alginate: 208.1	PLGA-CS: 23.79 PLGA-Alginate: −38.85,	Ac-PLP-BPI-NH_2_-2	-	-	Prophylactic: five days b.i. Preclinical/Therapeutic: four and 30 days p.i.	s.c.	300 nmol of colloidal gel per injection	Mice (6–8 weeks old) with EAE PLP139–151	A single injection of the colloidal gel containing the Ac-PLP-BPI-NH_2_-2 peptide led to long-term disease suppression.
**Soluble antigen arrays (SAgAs)**										
HA-peptide conjugate [209]	HA	-	PLP_139–151_	-	LABL, B7AP, CD80-CAP1, sF2 (cyclized)	Preclinical/Therapeutic: 4, 7, and 10 days p.i.	s.c.	200 nmol PLP peptide	SJL/J (H-2s) female mice (4–6 weeks old) with EAE induced with PLP_139–151_	SAgAs were shown to effectively reduce EAE incidence and suppress it via co-administration of an immunodominant myelin epitope and peptides targeting the B7 signaling pathway.
SAgAs [210]	HA	-	PLP_139–151_	-	LABL	Preclinical/Therapeutic: 4, 7, and 10 days p.i.	s.c.	200 nmol PLP_139–151_	SJL/J female mice (4–6 weeks old) with EAE induced with PLP_139–151_	Co-administration via conjugation of PLP_139–151_ and LABL improved the clinical scores of EAE
cSAgAs [184]	HA	-	PLP_139–151_		LABL	Preclinical/Therapeutic: 4, 7, and 10 days p.i.	s.c.	50, 133, or 200 nmol PLP_139–151_	SJL/J female mice (4–6 weeks old) with EAE induced with PLP_139–151_	cSAgAs was found to achieve equivalent efficiency with SAgAS regarding the suppression of EAE at a quarter of the SAgAS dose.
cSAgAs (Figure 11) [185]	HA	-	PLP_139–151_	-	LABL	Preclinical/Therapeutic: 4, 7, and 10 days p.i.	s.c.	50, nmol PLP_139–151_	SJL/J female mice (4–6 weeks old) with EAE induced with PLP_139–151_	Low dose s.c. vaccination with cSAgAS resulted in successful suppression of EAE clinical symproms and minimization of body weight loss.
SAgAs [210]	HA	-	PLP_139–151_	-	LABL	Preclinical/Therapeutic: 4, 7, and 10 days p.i.	pulmonary	65.1–74.5 mg SAgAs/mouse kg	Female SJL/J mice (four weeks old) with EAE induced with PLP_139–151_	The pulmonary administration of SAgAs was found to suppress the clinical score of the disease, decrease EAE incidence and improve weight gain.
SAgAs [183]	HA	-	PLP_139–151_	-	LABL	Preclinical/Therapeutic: 4, 7, or 10 days p.i.	i.p., upper and lower i.m., upper and lower s.c., i.v. pulmonary	200 nMol PLP per 100 μL injection volume 200 nMol PLP per 50 μL injection volume	Female SJL/J mice (6–8 weeks old) with EAE induced with PLP_139–151_	i.v. administration demonstrated similar efficiency with the other routes. p.i. vaccination decreased completely clinical disease scores. Single injection-based treatment resulted in decreased efficiency compared with a triple injection treatment. Decrease of SAgAs dose and/or injection volume decreased the therapeutic efficiency.
**Immune polyelectrolyte multilayers (iPEMs)**										
iPEMs [186]	-	-	MOG-R3	28.4–89.7%	GpG (0.7–10.3%)	Preclinical/Therapeutic: 5 and 10 p.i.	s.c.	200 μg of (MOG-R3/GpG)3 iPEMs, per injection.	C57BL/6J mice with EAE induced with a myelin antigen	s.c. delivery of iPEMs restrained inflammation and promoted autoimmune tolerance in an EAE mouse models.
iPEMs [187]	114.9–199.2	−42.5 to 33.4	MOGR1, MOGR2	0.57–9.18 μg of MOGRx	GpG 2.18 μg–4.88 μg	Preclinical/Therapeutic: seven days or 6, 12, and 18 days p.i.	s.c.	200 μg MOGR2 (85.9 μg GpG)	Female C57BL/6J mice (10 weeks old) with EAE induced with MOG_35–55_	iPEMs were shown to improve the severity, progression and incidence of EAE.
**Inorganic particles and pMHC-nanoparticles (pMHC-NPs)**										
Quantum dots [211]	15.0–21.0	−17.6 to −4.2	MOG	Up to 55	-	Preclinical: two days p.i.	s.c.		Female C57BL/6 mice (10–12 weeks old)	Ten-fold reduction of EAE incidence. Increased numbers of QDs with lower peptide loading were more efficient regarding the induction of immune tolerance.
Iron oxide NPs [212]	-	-	MOG_38–49_	-	IA^b^	Therapeutic: 14 or 21 days p.i.			C57BL/6 mice with EAE induced with pMOG_35–55_	By administration on day 14 the NPs were found to diminish the progression of the disease, whereas when administered on day 21 they were shown to restore the motor function of paralytic mice.
Iron oxide NPs [212]	-	-	hPLP_175–192_ hMOG_97–108_	-	DR4-IE	Therapeutic:			*HLA-DR4-IE*-transgenic C57BL/6 *IAb*null mice	Successful EAE suppression was observed.
Pegylated gold NPs [213]	60	-	MOG_35–55_ PLP_139–151_ PLP_178–191_		AhR ligand ITE	Prophylactic: admin. on the day of EAE induction Therapeutic: Admin. on day 17 post immunization. Weekly treatment of mice	parenteral	6 μg NPs per mouse	B6 mice with EAE induced with MOG_35–55_ SJL mice with EAE induced with EAE induced with PLP_139–151_	Pegylated gold NPs loaded with MOG35-55 and ITE significantly suppressed the development of EAE, whereas those loaded with PLP epitopes reduced the clinical scores of the disease and the number of relapses.
**Mannan-conjugated myelin peptides**										
Mannan-peptide conjugates (Figure 12) [188]	-	-	MOG_35–55_, PLP_139–151_, PLP_178–191_, MBP_83–99_	-	-	Prophylactic: 45, 30, and 15 days b.i. Preclinical/Therapeutic: Admin. on day 0 and 7 p.i.	i.d.	30 μg peptide/injection 700 μg mannan/injection	C57BL/6 mice (12–14 weeks old) with EAE induced with MOG Female SJL/J mice (6–8 weeks old) with EAE induced with PLP.	Mannan-peptide conjugates were shown to generate robust antigen-specific protection of mice from the clinical disease symptoms.
Mannan-peptide conjugates [214]	-	-	Linear and cyclic MBP_83–99_ peptide analogues cyclo(83-99)[A91]MBP83-99 mutant peptide			Preclinical/Therapeutic: Admin. on day 0 and 14 p.i.	i.d.	50 μg of linear and cyclic MBP_83–99_ peptide analogues	Female SJL/J mice (6–8 weeks old) with EAE induced with linear and cyclic MBP_83–99_ peptide analogues	It was shown that the mutant peptide cyclo(83–99)[A91]MBP_83–99_ more efficiently inhibited EAE development.
**Liposomes**										
Liposomes [190]	861.3	−36.2	MOG_40–55_	-/91.5	-	Preclinical/Therapeutic: 5 and 9 days p.i.	i.p.	1.75 mg of lipid per injection	C57BL/6 female mice (8 weeks old) with EAE induced with MOG_40–55_	Liposomes successfully delayed the onset, suppressed the severity and decreased the incidence of the disease.
(mannosylated) SUV [189]	~85	−7.5 to −10.5	MBP_46–62_ MBP_124–139_ MBP_147–170_	-/90	-	Preclinical/Therapeutic: admin. on day 7 post immunization followed by five consecutive days.	s.c.		Female DA rats (8–9 weeks old) with EAE induced with a syngeneic spinal cord homogenate or with MBP_63-81_.	It was revealed that mSUVs loaded with immunodominant epitopes of MBP could significantly suppress EAE in DA rats.
**Exosomes**										
mTGF-β1-EXOs [215]	50–100					Prophylactic: 8, 5, and 2 days b.i. Therapeutic: 14, 17 and 21 days p.i.	i.v.	10 μg/mouse/injection	Female C57BL/6 mice (6–8 weeks) with EAE induced with MOG_35–55_ Female BALB/c mice (6–8 weeks) with EAE induced with PLP_180-199_	Treatment with mTGF-β1-EXOs from C57BL/6 mice successfully inhibited the development and progression of the disease in both mice strains.
**Antigen-presenting yeast cells**										
*C. utilis* expressing MOG_35–55_ on its surface [216]	-	-	MOG_35–55_ pCB13 pCB10			Prophylactic: admin. on day 7 prior to immunization and for six consecutive days	Oral	1.5 × 10^8^ *C. utilis*	Female C57BL/6 mice (eight weeks old) with EAE induced with MOG_35–55_	*C. utilis* expressing MOG_35–55_ on its surface appeared to be a promising approach to protect myelin against autoimmunity by effectively inducing oral tolerance. Fungal viability was not found to affect the induction of tolerance.

PLGA: poly(lactide-co-glycolide); NPs: nanoparticles; MOG: myelin oligodendrocyte glycoprotein; (r) IL-10: recombinant interleukin; s.c.: subcutaneous; b.i.: before immunization; p.i.: post immunization; EAE: experimental autoimmune encephalomyelitis; PLP: proteolipid protein; TGF-β: transforming growth factor beta 1; i.v.: intravenous; MPs: microparticles; GM-CSF: granulocyte-macrophage colony-stimulating factor; PEMA: poly[ethylene-alt-maleic anhydride]; i.p.: intraperitoneal; PEG: polyethylene glycol; PLA: polylactide; PEI: polyethylene imine; Ac-PLP-BPI-NH2-2: (Ac-HSLGKWLGHPDKF-(AcpGAcpGAcp)2-ITDGEATDSG-NH2; Ac = acetyl, Acp = aminocaproic acid); CS: chitosan; SAgAs: soluble antigen arrays; HA: hyaluronic acid; LABL: ICAm-I binding peptide; cSAgAs: Click Soluble Antigen Arrays; i.p.: intraperitoneal; i.m.: intramuscular; iPEMs: immune polyelectrolyte multilayers; GpG: GpG oligonucleotide; MOGR3: MOG conjugated to tri-arginine; MOGR1 and MOGR2: MOG modified with either one or two cationic arginine residues; SUV: small unilamellar vesicles; mTGF-β1-EXOs: exosomes from dendritic cells expressing membrane-associated TGF-β1.

**Table 6:** Clinical trials.

Objective	Phase	No. of Particip.	Antigen Immunotherapy	Admin. Route/Dose/Duration of Treatment	Results
To suppress disease activity in RRMS patients using CGP77116 [74]	II	24	CGP77116	s.c. injection/50 mg CGP77116 per week; 5 mg per week; 5 mg per month/9 months	Decrease of dose because of adverse effects. Trial termination due to treatment-related disease exacerbation.
Evaluation of NBI 5788 safety, and effect on RRMS patients [217]	II	144	NBI5788	s.c. injection/5, 20, or 50 mg NBI5788 per week/4 months	Trial suspension due to hypersensitivity reactions in some patients. No increase in relapses. Reduction of number and volume of enhancing lesions in patients who completed the trial receiving 5 mg of NBI5788 per week.
Assessment of safety, tolerability and clinical activity of AG284 in SPMS patients [218]	I	33	AG284	/0.6, 2, 6, 20, 60, 105, and 150 mg AG284/kg body weight; each dose was received daily for three alternate days/	No adverse events but also no significant therapeutic effect could be observed.
Assessment of the clinical efficiency of MBP_82-98_ in patients with progressive MS [219]	II	32	MBP_82–98_	i.v./500 mg MBP_82-98_ per 6 months/24 months	Only patients with HLA haplotypes DR2 and/or DR4 appeared to have benefited from the treatment.
Evaluation of the safety and efficiency of MBP_82-98_ in SPMS patients with HLA haplotypes DR2 and/or DR4 [220]	III	612	MBP_82–98_	i.v./500 mg MBP_82-98_ per 6 months/2 years	The administration of was safe and well tolerated. The treatment was not effective in SPMS patients with HLA DR2^+^ or DR4^+^
Evaluation of RTL1000 safety in MS patients [221]	I	34	RTL1000	i.v./2, 6, 20, 60, 200, and 100 mg of RTL/	RTL1000 was safe at doses ≤ 60 mg
Determination of the maximum tolerable dose and safety of RTL1000 in MS patients [222]	I	36	RTL1000	i.v./2, 6, 20, 60, 200, and 100 mg of RTL/	The maximum tolerable dose of RTL100 was 60 mg.
Examination of the effect of high dose MBP_82-98_ on the number of regulatory T cells in CPMS patients [223]		10	MBP_82–98_	i.v./500 mg of MBP_82-98_ per 6 months/	Increase in the number of regulatory T cells in patients’ PBMCs six weeks and six6 months after treatment. Renversement of the state of T cell anergy.
Assessment of safety and tolerability of autologous PBMCs coupled with 7 myelin peptides in RRMS and SPMS patients [224]	I	9	PBMCs chemically coupled with the following 7 myelin peptides: MOG_1–20_, MOG_35–55_, MBP_13–32_, MBP_83–99_, MBP_111–129_, MBP_146–170_, and PLP_139–154_	Single infusion/1 × 10^3^, 1 × 10^5^, 1 × 10^7^, 1 × 10^8^, 1 × 10^9^, 2.5 × 10^9^ and 3 × 10^9^ antigen-coupled PBMCs/3 months	The treatment was found to be safe and well-tolerated. Antigen-specific T cell responses were shown to decrease after treatment in patients who received doses ≥1 × 10^9^ of antigen coupled PBMCs.
Examination of BHT-3009 safety and feasibility for immune nodulation in RRMS and SPMS patients [225]	I/II	30	BHT-3009	i.m./0.5, 1.5, and 3 mg of BHT-3009 at weeks 1, 3, 5, and 9 after patients’ randomization into the clinical trial/The administration of BHT-3009 was combined or not with daily oral administration of 80 mg atorvastatin.	BHT-3009 was found to be safe and to induce antigen-specific immune tolerance in MS patients. The co-administration of atorvastatin was not considered substantially beneficial.
Assessment of the transdermal delivery of a mixture of three myelin peptides to induce immune tolerance in RRMS patients [226]		30	Mixture of the following 3 myelin peptides: MBP_85–99_, PLP_139–151_, and MOG_35–55_	Transdermal (via an adhesive skin patch)/1 or 10 mg of each myelin peptide per week (for 4 weeks) and per month (for 11 months)/1 year	The transdermal administration of myelin peptides was proven to be tolerogenic in RRMS patients.
Assessment of safety and efficiency of transdermal administration of myelin peptides in RRMS patients [227]		30	Mixture of the following three myelin peptides: MBP_85–99_, MOG_35–55_, and PLP_139–151_	Transdermal (via an adhesive skin patch)/1 or 10 mg of each myelin peptide per week (for four weeks) and per month (for 11 months)/1 year	The transdermal delivery of myelin peptides was found to be safe, well tolerated and to reduce clinical symptoms and number of Gadolinium lesions in RRMS patients.
Evaluation of BHT-3009 regarding its safety and efficiency to induce immune tolerance in RRMS patients [228,229]	II	289	BHT-3009	i.m./ 0.5 and 1.5 mg of BHT-3009 at weeks 0, 2, 4, and every four weeks until week 44/The administration of BHT-3009 was combined or not with daily oral administration of 80 mg atorvastatin.	It was shown that treatment with the lower dose of BHT-3009 (e.g., 0.5 mg) succeeded in inducing antigen-specific immune tolerance in some patients in contrast with the higher dose (e.g., 1.5 mg) which was found to be ineffective.
Evaluation of ATX-MS-1467 safety in SPMS patients [117]	I	6	ATX-MS-1467	i.d/25, 50, 100, 400, and 800 μg of ATX-MS-1467/	The safety and tolerability of ATX-MS-1467 at a dose ≤ 800 μg, was successfully demonstrated in SPMS patients.
Evaluation of ATX-MS-1467 safety, tolerability and efficiency to induce tolerance in RRMS patients [230]	Ib, IIa	43, 37	ATX-MS-1467	Ib: i.d. (cohort 1) or s.c. (cohort 2)/25, 50, 100, 400 and 800 μg of ATXMS-1467 per two weeks (for eight weeks) and 800 μg per two weeks (for eight more weeks)/one year (including 32 weeks medication off study). IIa: i.d./50 μg of ATXMS-1467 (on day 1), 200 μg (on day 15), 800 μg (on day 29), and 800 μg per two weeks (for 16 more weeks)/one year (including 16 weeks medication off study).	Both treatment protocols were found to be safe. The relatively slow i.d. titration of ATX-MS-1467 followed by a longer high dose treatment period resulted in reduced GdE lesions which remained so even post treatment.
**Tolerogenic DCs (tolDCs)**					
Evaluation of the safety of myelin peptide loaded tolDCs and their ability of to induce immune tolerance in MS patients. [231]	I	8	Autologous tolDCs loaded with myelin peptides	i.v./50 × 10^6^, 100 × 10^6^, 150 × 10^6^, and 300 × 10^6^ tolDCs divided in three independent doses administered every two weeks/	Myelin peptide loaded tolDCs were proven to be safe and well tolerated, and to induce tolerogenic responses in MS patients.
Evaluation of the safety of intradermal and intranodal delivery myelin peptide loaded tolDCs and their efficacy regarding the induction of antigen-specific tolerization in MS patients [232]	I	9–15	Autologous peptide-mix loaded tolDCs	i.d. or intranodal/six repetitive doses of 5 × 10^6^, 10 × 10^6^ and 15 × 10^6^ autologous peptide-mix loaded tolDCs: administration of doses 1–4 once every two weeks and of doses 5–6 once every month.	-
**T-cell vaccination (TCVs)**					
Assessment of safety and immune efficiency of a polyclonal T cell vaccine in chronic MS patients in advanced diseases stages [233]		39	autological polyclonal TCVs	s.c./1.5–3 × 10^7^ polyclonal T cells; four weekly injections followed by monthly injections.	Polyclonal TCV was proven safe and capable of inducing long-lasting, anti-inflammatory immune effects in progressive MS patients in advanced disease states.
To establish a safe and efficient dose of Tovaxin^®^ [234]		9–15	Attenuated T cells reactive to the following myelin peptides MBP_83–99_, MBP_151–170_, PLP_30–49_, PLP_180–199_, MOG_1–17_ and MOG_19–39_	s.c./6–9 × 10^6^, 30–45 × 10^6^, and 60–90 × 10^6^ administered at weeks 0, 4, 12, and 20/	The study indicated the mid-dose as optimum with respect to safety, and efficiency in reducing peripheral blood myelin reactive T cells and showing a trend to improve clinical symptoms.
Evaluation of safety and efficacy of Tovaxin in RRMS patients [235]	IIb	150	T cells reactive to different immunodominant peptides from three myelin proteins	s.c./five injections at weeks 0, 4, 8, 12, and 24	s.c. administration of Tovaxin was shown to be safe. Evidence of clinical efficiency of Tovaxin^®^ was observed during the analysis of subgroups of patients naïve to prior disease modifying therapies.
Examination of TCV safety and efficiency in progressive MS patients [236]	II	26	T-cell lines reactive to nine different peptides of MBP, MOG and PLP.	19 patients received s.c. TCV/10–30 × 10^6^ T cells, on days 1, 30, 90 and 180/7 patients received sham injections.	The clinical trial demonstrated the safety of TCV in progressive MS patients and indicated its clinical efficiency.
Assessment of TCV safety and immune modulation in RRMS and CPMS patients [237]	pilot	5	CSF derived activated CD4+T cells	3 s.c. injections; 10^6^ cells at months 2, 4, and 6.	TCV was safe and well tolerated. Patients were clinically stable or exhibited reduced EDSS without relapses during and post treatment.
Examine if the depletion of T cells reactive to MBP would have a clinical benefit for RRMS and SPMS patients [238]	Preliminary	54	Irradiated autologous T cells reactive to MBP-	3 s.c. injections at 2 month intervals, 30 × 10^6^–60 × 10^6^ cells per injection.	A 40% decrease in the relapses rate and a minimal decrease in EDSS was observed in RRMS patients. On the other hand, a slight increase of EDSS was detected in SPMS patients. Finally, MRI scans indicated a stabilization of the lesion activity.
Assess the use of T cell lines reacting with a broad range of antigens regarding targeting and depletion of specific T cells reactive to a great number of myelin antigens in SPMS patients. [239]	Pilot	4	Peripheral blood derived T cell lines reactive to bovine myelin		TCV with T cells reactive to whole bovine myelin were shown to efficiently promote depletion of circulating T cells reactive to myelin protein.
Evaluation of the TCV efficiency in patients with aggressive RRMS non-responding to DMTs [240]		20	Autologous attenuated T cell lines reactive to MBP and MOG encephalitogenic peptides.	Three s.c. injections in six- to eight-week intervals.	TCV was proven to be safe. A decrease in the relapse rate was observed. Additionally, significant decrease in the active lesions regarding number and volume as well as in T2 lesion burden was detected.
Identification of the idiotypic determinants triggering CD81 cytotoxic anti-idiotypic responses by TCV in MS patients [241]		3	Irradiated autologous T cell clones reactive to MBP_83–99_	s.c./repetitive injections of 2 × 10^7^ of each cell clone every 2 months for 8 months.	CD3-specific T cells were recognized as a representative anti-idiotypic population of T cells induced by TCV.
**T-cell receptor (TCR)**					
To examine the therapeutic potential of a trivalent TCR vaccine in MS patients [242]		23	A trivalent TCR vaccine containing the CDR2 peptides BV5S2, BV6S5 and BV13S1	12 monthly vaccinations	The therapeutic TCR vaccine induced an extended immunoregulatory network which could control complex self-reactive responses of MS.
**Liposomes**					
Assessment of Xemys safety and efficiency in treating RRMS and SPMS patients non-responding to DMTs [191,192]	I	20	Xemys: Liposomes loaded with MBP_46–62_, MBP_124–139_ and MBP_147–170_ And targeting CD206	s.c./six weekly injections of 50, 150, 225, 450, 900, and 900 μg Xemys	The administration of Xemys was proven to be safe and well tolerated, and to normalize cytokine levels in RRMS and SPMS patients.

RRMS: relapsing remitting multiple sclerosis; CGP77116: APL of MBP_83–99_; APL: antigen peptide ligand; MBP: myelin basic protein; s.c.: subcutaneous; NBI 5788: APL of MBP_83–99_; AG284: solubilized complex of HLA-DR2 with MBP_84–102_; HLA: human leucocyte antigen; SPMS: secondary progressive multiple sclerosis; i.v.: intravenous; RTL1000: recombinant T-cell receptor ligand 1000; CPMS: chronic progressive multiple sclerosis; PBMCs: peripheral blood mononuclear cells; BHT-3009: tolerizing DNA vaccine encoding MBP; i.m.: intramuscular; ATX-MS-1467: mixture of equal quantities of synthetic peptides ATX-MS1 (MBP_30–44_), ATX-MS4 (MBP_131–145_), ATX-MS6 (MBP_140–154_), and ATX-MS7 (MBP_83–99_) in PBS; PBS: phosphate- buffered saline; i.d.: intradermal; tolDCs: tolerogenic dendritic cells; Tovaxin^®^: autologous T-cell immunotherapy; MOG:, PLP:; CSF:; DMTs: disease modifying therapies; CDR2: complementarity determining region 2.
